# Shotgun metagenomic sequencing analysis of ocular surface microbiome in Singapore residents with mild dry eye

**DOI:** 10.3389/fmed.2022.1034131

**Published:** 2022-11-10

**Authors:** Louis Tong, Florentin Constancias, Aihua Hou, Song Lin Chua, Daniela I. Drautz-Moses, Stephan Christoph Schuster, Liang Yang, Rohan B. H. Williams, Staffan Kjelleberg

**Affiliations:** ^1^Ocular Surface Research Group, Singapore Eye Research Institute, Singapore, Singapore; ^2^Corneal and External Eye Disease Service, Singapore National Eye Centre, Singapore, Singapore; ^3^Eye-Academic Clinical Programme, Office of Clinical, Academic and Faculty Affairs, Duke-NUS Medical School, Singapore, Singapore; ^4^Department of Ophthalmology, Yong Loo Lin School of Medicine, National University of Singapore, Singapore, Singapore; ^5^Singapore Centre for Environmental Life Sciences Engineering (SCELSE), Nanyang Technological University, Singapore, Singapore; ^6^Lee Kong Chian School of Medicine (LKCMedicine), Nanyang Technological University, Singapore, Singapore; ^7^School of Biological Sciences, Nanyang Technological University, Singapore, Singapore; ^8^School of Medicine, Southern University of Science and Technology, Shenzhen, China; ^9^Life Sciences Institute, National University of Singapore, Singapore, Singapore; ^10^Centre for Marine Science and Innovation, School of Biological Earth and Environmental Sciences, University of New South Wales, Kensington, NSW, Australia

**Keywords:** human microbiome, inflammation, ocular disease, dry eye, metagenomics

## Abstract

The ocular surface microbiome has implications for ocular surface inflammation and immunology. Previous shotgun metagenomics analyses were performed in China, showing results that differed according to environment and age. Patients with Sjogren’s syndrome were reported to have altered conjunctival microbiome, but such studies have not been done in milder dry eye. The aim of this study is to describe the conjunctival microbiome in people with mild dry eye in Singapore. Samples were collected from 14 participants with mild dry eye and 10 age-matched comparison participants recruited from Singapore National Eye Centre (SNEC) clinics. Shotgun metagenomic sequencing analysis was employed to evaluate the conjunctival microbiome composition. *Proteobacteria* formed the predominant phylum in the conjunctiva. As in a study from a coastal city in China, *Achromobacter* spp. was numerically most abundant. Compared to age-matched controls, the conjunctival microbial composition in mild dry eye was similar. Several microorganisms, including *Streptococcus* spp. increased in representation with age, and the abundance of *Staphylococcus* correlated with Schirmer readings. In addition, when cultured corneal epithelial cells were exposed to three strains of *Achromobacter xylosoxidans*, cytokines such as TNF-α and IL-6 were upregulated in the cell lysates and supernatants. Ourresults suggest that age is an important factor that affects composition of the conjunctival microbiome, and relative abundance of specific microorganism may vary according to the environment of the human host.

## Introduction

The human ocular surface consists of the conjunctiva, corneal surfaces, the lid margin, tear secreting glands and outflow tracts. The ocular surface microbiome is important for several reasons. First, it has been linked to various human eye diseases such as dry eye (1–4), Sjogren’s syndrome (5, 6), allergic conjunctivitis (7, 8), trachoma (9) and infectious keratitis (10, 11). Second, microbial components in the eye are known to stimulate toll-like receptors on the ocular surface epithelial cells, thereby activating the ocular surface immune system (12–15). The conjunctival-associated lymphoid tissue and lymphatics in the conjunctiva (16–19) may play an analogous role to the Peyer’s patches in the small intestine for the regulation of immune tolerance (20–23). Topical probiotic eyedrops containing *Lactobacillus acidophilus* reduces severity of ocular allergies (24) in humans, while the use of topical eyedrops with *Enterococcus faecium* and *Saccharomyces boulardii* showed improvement in signs of dry eye in a clinical trial (25), suggesting that modulation of ocular surface inflammation *via* microbial strategies is possible. Furthermore, an oral probiotic formulation has been shown to reduce severity of experimental dry eye (26–28). Third, the role of ocular surface microorganisms in eyelid conditions like blepharitis and eye infections is well documented (29).

Conventional culture-based studies of the ocular surface have revealed a predominance of Gram-positive cocci such as coagulase-negative *Staphylococci*, Diphtheroids, and anaerobes, including *Propionibacter acnes* (30–32). Studies with 16S rRNA amplicon sequencing have reported a slightly different community profile, with more Gram-negative bacteria (4, 9, 33–35). Although 16S rRNA amplicon-based microbiome studies allowed a broad description of bacterial community, this method has low phylogenetic resolution at the species level (36). Shotgun metagenomic analysis sequences the whole community DNA, and allows the community profiling of the bacteria to species level (37–41). Furthermore, whole genome information enables analyses of metabolic and functional pathways. One such study reported that pathways related to transcription, lipid and amino acid metabolism were abundant in the healthy ocular surface microbiome (37). Previous studies using shotgun metagenomic methods showed that environmental factors impact healthy ocular surface microbiome (38). Since the previous studies using shotgun metagenomics were from China, it is necessary to replicate them in a different climate.

As inflammation is the most common cause of ocular surface disease, understanding how microbiomes relate to different types of inflammation is necessary for effective treatment. There are implications for clinical practice in ocular allergies, eyelid-induced ocular surface inflammation, contact lens wear, extended corticosteroid use, as well as prophylaxis against iatrogenic infections related to surgeries (31, 35, 42). It is known that SS patients have altered ocular surface microbiome, but this has not been studied in mild dry eye.

This study aimed to describe ocular surface microbiome in a group of people with mild dry eye and comparison participants living in Singapore, explore its association with demographic and parameters related to ocular surface health. In order to understand effect of the microbes to ocular surface inflammation, the impact of preponderant microbial species on cytokine expression by ocular surface epithelial cells was investigated.

## Materials and methods

### Participant recruitment

The study was approved by the SingHealth centralized Institutional Review Board, and complied with the tenets of the Declaration of Helsinki for human research. Informed written consent was obtained from all participants. Participants who had been diagnosed to have dry eye had a spectrum of results on clinical tests, with presence of dry eye symptoms and one clinical sign (either Schirmer I test results < 10 mm or NIBUT < 10 s or presence of corneal staining). Supplementary Table 1 showed clinical features of the participants. These dry eye cases were referred to as mild because all participants were level 1 except one with level 2 in the DEWS 2007 severity criteria (43). Participants with diabetes were excluded because they could have altered microbiomes (44, 45). Participants in the comparison group did not satisfy the above dry eye criteria and presented to Singapore National Eye Centre (SNEC) for an unrelated eye condition such as floaters.

The participants were evaluated with a questionnaire that identified risk factors of dry eye such as contact lens use, smoking (46). The use of artificial tears and contact lens wear are provided in Supplementary Table 2. None of the dry eye participants had treatment with prescription eye drops (corticosteroids, cyclosporine or antibiotics). The control group had no topical treatment including artificial tears and antibiotics, and no contact lens wear for the past 6 months.

### Clinical examination

Keratograph (K) 5M (Oculus Inc., Arlington, WA) was used to assess the non-invasive tear breakup time (NIBUT). The machine’s algorithm monitored the tear film to document the time and site of tear film breakup. This technique analyzed reflection images of Placido rings at intervals after eye opening, to measure the smoothness of the tear-air interface. Tear irregularity presents as disruption of the images of the rings. A lower value of NIBUT indicates increased tear evaporation or greater tear instability.

Bulbar conjunctival and peri-limbal eye redness was also assessed by K5M (47). An image of the ocular surface under white light was acquired with the K5M and automatically given a standard grading for conjunctival hyperemia, ranging from 0 (no redness) to 4.0 (maximum redness). This analysis also provided separate measures of redness of the temporal and nasal conjunctiva.

Baseline tear secretion was measured by Schirmer’s test without anesthesia as described previously (48). A lower Schirmer I value indicates less tear secretion. The corneal fluorescein staining was performed and graded as previously described (46, 49).

### Specimen collection

A drop of non-preserved tetracaine was firstly instilled into the conjunctival fornix. After the stinging sensation had resolved, a sterile cotton swab was used to collect the microbes from the lower conjunctival fornix using a gentle rolling action (up to eight strokes). The procedure was then repeated for the opposite eye. The cotton swabs from both eyes were combined and then soaked in 650 μl of DNA/RNA Shield (Zymo Research Corp., Irvine, CA) reagent, immediately homogenized for 30 s, transferred to ice for 1 min, and further homogenized for another 30 s. Homogenized samples were stored at 4°C until further processing (within 1 week). Total DNA was extracted with ZR-Duet DNA/RNA MiniPrep (Zymo Research, Irvine, CA). Empty swabs following the same procedure were used as control. An empty swab was an unused swab that was opened under the same room and conditions as the participants and then homogenized and processed as if it has been used on a participant.

### Metagenomic library preparation and sequencing

DNA quality and quantity were determined using a 2100 Bioanalyzer and the Invitrogen PicoGreen assay, respectively. Library preparation was performed according to the Illumina TruSeq Nano DNA Sample preparation protocol. The samples were sheared on a Covaris S220 (Covaris, Woburn, MA, USA) to ∼450 bp, following the manufacturer’s recommendation, and each uniquely tagged with one of Illumina’s TruSeq LT DNA barcodes. Sequencing was performed on the Illumina HiSeq 2500 platform (Illumina, San Diego, CA, USA) resulting in an average of 56.18 million (49.29–77.79 M) 250 bp paired-end reads per sample. Sequence data were deposited in the Sequence Read Archive and are available under the BioProject PRJNA886972.

### Read preprocessing

Illumina adaptors were removed using cutadapt (version 1.10). Low-quality reads were removed using the program “iu-filter-quality-minoche.” Overall, quality-trimmed reads represented 83% (81–86%) of DNA reads. Human reads were then removed from the dataset by aligning DNA reads to the human genome (h38 from NCBI: GCF_000001405.36_GRCh38.p10_genomic.fna.gz) using Bowtie2 (version 2.2.9). Overall, around 0.11 million (0.04–0.27 million) DNA reads were retained after quality filtering and removal of human reads.

### Taxonomic and functional profiling of the ocular microbiome

Taxonomic composition of DNA reads was characterized by alignment against the NCBI non-redundant (NR) protein database (March 2016)^1^ using DIAMOND (version 0.7.10.59) with default parameters. The lowest common ancestor approach implemented in MEGAN6 (version CE_6_5_5, -ms 100 -supp 0 –sup 25 –pr –ps 2) was used to assign reads at the phylum, genus and species levels. Each aligned read was assigned a KEGG KO number using KEGG to GI mapping file generated using KEGG 01/04/2016 repository according to MEGAN manual.

### Statistical analysis

Phylum, genus, species and KEGG count tables from the metagenomic dataset were exported from MEGAN6 and imported in R using the phyloseq package. Count tables were filtered to remove taxa/KO accounting for less than 10 sequences in total and observed in less than two samples (*filter_taxa* function). Taxonomic and functional tables were then rarefied to an even sequencing depth using the *rarefy_even_depth* function to allow robust comparison between samples. Microbial communities were characterized using alpha-diversity indices (number of observed taxa/K0 and Shannon diversity indices) and beta-diversity (Bray-Curtis dissimilarity) for taxonomic (phylum, genus and species levels) as well as functional datasets.

In order to investigate the correlation between microbial communities and ocular parameters, distance-based redundancy analysis models (db-RDA) were conducted between scaled ocular parameters and taxonomical or functional Bray-Curtis dissimilarities. The statistical significance was assessed by 999 permutations of the reduced model. Spearman correlations were conducted between ocular parameters and taxa/KO. Statistical significance of taxa/KO-group association was tested using the “signassoc function” from the “indicspecies” package. Sidak’s correction was applied for multiple testing.

### Cell culture and treatment

*Achromobacter xylosoxidans* strains were obtained from ATCC (DSMZ, Braunschweig, Germany). Human corneal epithelial cells (H-CET) were cultured to near confluency in DMEM/F12 medium containing 5% FBS (Life Technology, Singapore) media, with passaging and maintenance of culture conditions as described in previous studies (50). Before experiments, cells were washed, seeded into serum-free DMEM/F12 medium and grown overnight.

For treatment experiments, the following conditions were tested in triplicates: (1) DMEM only: H-CET control; (2) DMEM + 1μg/ml LPS: H-CET activated with 1μg/ml LPS (Sigma) for 16 h; (3) *Achromobacter xylosoxidans* strain 1 or 2 or 3 + 1μg/mL LPS: H-CET activated with 1μg/mL LPS for 16 h, followed by infection of 1:100 MOI of the strain of *Achromobacter* for 2 h. The extracellular bacteria were then removed by washing three times with PBS and the H-CET were then incubated in DMEM for another 3 h; (4). The same conditions as in (3) were investigated, but without the addition of the LPS.

### Cytokine analysis

The cell culture media and cells were collected separately for analysis. As for the cell culture media supernatant, the liquid was transferred to a new 1.5 ml Eppendorf tube, centrifuged at 13,000 g for 3 min. The supernatant was then filtered with 0.2 μm-filter. The cells were washed three times with 200 μL PBS/well, lysed by addition of 200 μL of ddH_2_O/well and scraped from the bottom of the well with a cell scraper. The cell lysates were transferred to a new 1.5 mL Eppendorf tube, centrifuged at 13,000 g for 3 min. The supernatant was then filtered with the 0.2 μm-filter.

Cell lysate protein concentrations were determined by the bicinchoninic acid method. The same volume of supernatant (25μL/well) and same amount of cell lysate proteins (29.5μg/well) from each sample were used for multiplex bead-based indirect immunofluorescent assay (Beadlyte; EMD Millipore, Billerica, MA, USA) as described previously (51). Each sample was triplicated. Levels of 15 cytokines (IL-1β, IL-2, IL-4, IL-6, IL-8, IL-10, IL-12, IL-13, IL-17, IFN-γ, TNF-α, IP-10, MCP, MIP1a, RANTES) were analyzed.

## Results

There were no significant differences in age, gender or ethnicity of participants with dry eye and controls (Table 1). The dry eye participants recruited in this study were mainly mild in severity, with presence of dry eye symptoms and one clinical sign (either reduced Schirmer I test results or NIBUT or presence of corneal staining). Since the participants had an abnormal result in only one out of these tests, each test displayed a wide range of readings across the entire group (Table 1). Among the ocular surface clinical parameters, the extent of conjunctival redness was directly correlated to increased age. Age showed a bimodal distribution with one peak below and another above 40 years of age (Supplementary Figure 1).

**TABLE 1:** Clinical and demographic characteristics of participants.

	Overall	Control	Dry eye^ 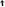 ^
Total number	24	10	14
% female	17/24	7/10	10/14
% Chinese	19/24	7/10	12/14
**Age (years)** *mean* ± *SD*	*44.2* ± *15.0*	*44.1* ± *14.3*	*44.3* ± *16.2*
**Redness (1–4)** *mean* ± *SD*	1.0 ± *0.4*	1.1 ± 0.4	1.0 ± 0.4
**Schirmer (mm)** *mean* ± *SD*	13.9 ± *8.7*	*14.0* ± *10.9*	*13.8* ± *6.2*
**NIBUT**^ 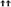 ^ **(s)** *mean* ± *SD*	*9.3* ± *5.8*	*8.7* ± *5.2*	*9.9* ± *6.5*
**Fluorescein staining in any corneal zone** ^ 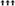 ^	8/24	3/10	5/14

^
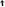
^Participants are classified under dry eye if they demonstrated dry eye symptoms and one of the clinical signs (Staining, Schirmer’s test or NIBUT). Since participants in the dry eye group tend not to have abnormal results in all the tests: staining, Schirmer’s test and NIBUT, these parameters are individually not lower than the control group, and the values had a large SD. ^
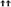
^Non-invasive tear break up times. ^
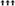
^The staining were mild in the zones when present, with no cases of above 10 fluorescein spots in any single corneal zone. Control and dry eye groups were not significantly different in any of the parameters above (*p* > 0.05).

### Characterization of the microbiome

Sequencing reads were obtained in each of the 24 samples, as summarized in Table 2. Of 56,181,504 raw reads, most of the reads were human in origin and 1,246,495 were matched to identified microbial phyla. The top major phyla identified in these participants are shown in Supplementary Figure 2. The phylum *Proteobacteria* represents the most abundant phylum, followed by *Bacteroidetes*. The most common fungus phylum was *Basidiomycota* (which includes free living organisms and *Cryptococcus).* The bacterial phylum *Firmicutes* (which includes the *Clostridia, Streptococcus, Staphylococcus and Lactobacillus*) was significantly over-represented among the older (>40 years of age), compared to younger (<40 years of age) participants (Supplementary Figure 3). Bacterial reads outnumbered fungal and viral reads (data not shown).

**TABLE 2:** Sequencing reads.

	Mean ± SD	Min	Max
Raw	56,181,504 ± 5,958,525	49,290,548	77,791,732
Passing QC	54,721,178 ± 5,692,465	47,637,514	76,179,154
Non-human reads	315,589 ± 98,432	181,418	545,159
Final matches	1,246,495 ± 395,038	508,348	2,014,630
Queries aligned	56,512 ± 17,717	23,209	91,073
Class taxonomy	1,375 ± 5,855	101	28,864
Class KEGG	2,363 ± 511	260	2,772
Total reads (%)	31.4 ± 9.8	12.9	50.6

The most abundant genus was *Achromobacter*, with the most common species identified as *Achromobacter xylosoxidans* (Supplementary Figure 4). There were no significant differences in abundance of any microbial genus, or species between dry eye and control participants, among the top 10 genera and species. The most abundant phylum and genus from the empty swabs control are showed in Supplementary Figure 5. *Achromobacter* was not among the top 10 genera in empty swab control samples.

**FIGURE 1:**
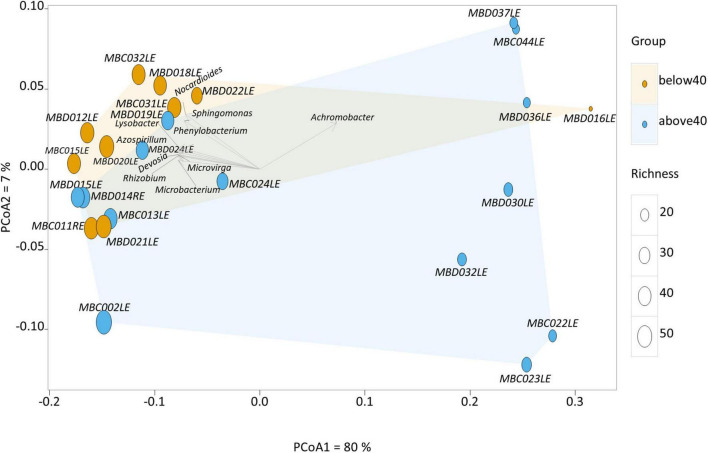
Principal component analysis of the human conjunctival microbiome composition. This scatter diagram shows each participant (symbol) along the first two principal components of the microbial gene analysis at the genus level. The age of the participant is displayed as either orange (younger) or blue (older), and richness is displayed as differing sizes of the symbols. Certain bacterial genera contribute significantly to loading scores in the first principal component (horizontal axis) illustrated in the scatter diagram.

**FIGURE 2:**
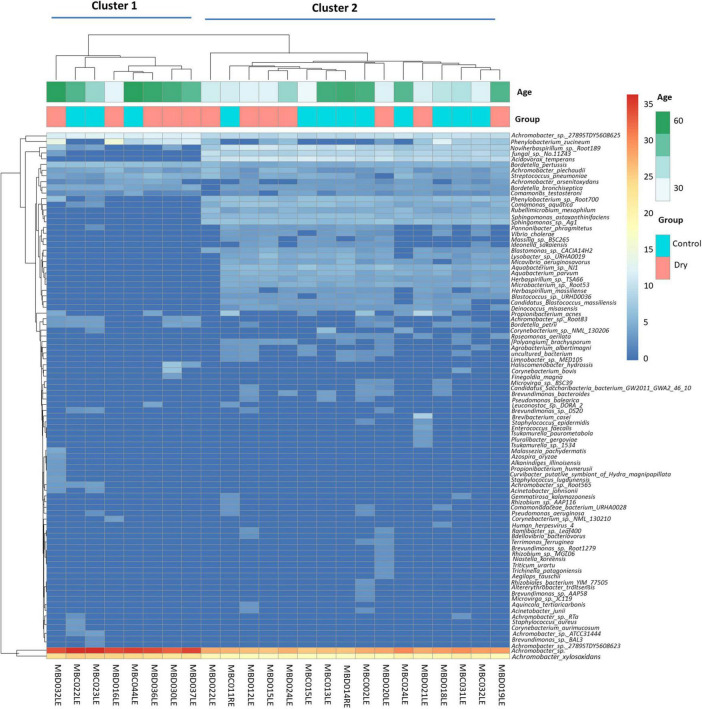
Heat maps showing hierarchical clustering of abundance of microbial species in the human conjunctival microbiome. This shows two distinct clusters (cluster 1: eight patients, and cluster 2: 16 patients). The identity of the species is indicated on the vertical axis and each column represents one participant. Horizontal axis annotation **(top)** shows the age of the participants and the dry eye category.

Two clusters of participants were identified using principal component analysis (Figure 1), one large cluster (16 participants) and a small but more dispersed cluster of eight participants (MBD016LE, MBD036LE, MBC044LE, MBD037, MBD030LE, MBC022L, MBC023LE, MBD032LE). There were no significant differences in the dry eye parameters between participants of the two clusters, although participants of the smaller cluster (*n* = 7) were of an older age (*p* = 0.028). The ages in the two clusters were 39.6 ± 13.6 years and 53.5 ± 13.7 years, respectively. The smaller cluster (Figure 2 left) had an increased representation of *Achromobacter* spp. and a reduced proportion of a number of microbial species (e.g., *Acidovorax temperans, Phenylobacterium zucineum, and Noviherbaspirillum* spp.) compared to the larger cluster (Figure 2 right). Supplementary Table 3 listed all the bacteria that are under-represented in the smaller cluster.

It is interesting to examine the bacteria under-represented in the smaller cluster in greater detail, as the age effect on this bacterial community may have physiological relevance, even though individual microbes may not play a significant role. On closer examination, this group of 23 bacterial species contain mainly bacteria from the phylum *Proteobacteria*, with the exception of four species. These exceptions were *Deinococcus misasensis*, *Rubellimicrobium mesophilum* and *Candidatus* Blastococcus massiliensis*;* a fourth species, *Micavibrio aeruginosavorus* which, is a known epibiotic obligate bacterial predator that feeds on potential disease-causing bacteria such as *Pseudomonoas aeruginosa.* A reduction in abundance of *M. aerugonosavorus* may have health effects, since the protective functions of *M. aeruginosavorus* may be reduced as its relative numerical composition is decreased.

### Association with clinical parameters

**FIGURE 3:**
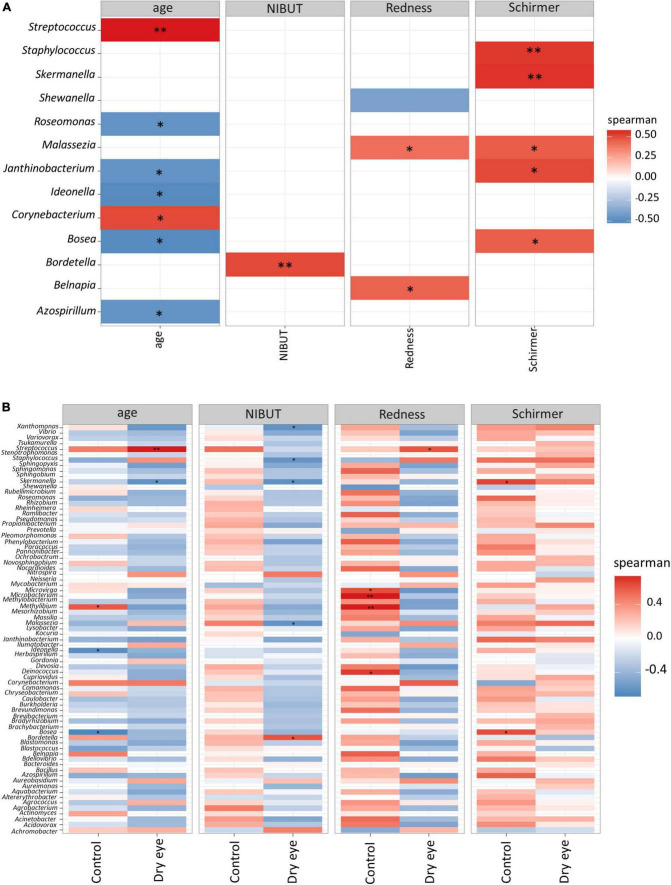
Correlation between microbial genera and demographic/clinical parameters. Each horizontal row indicates a unique microbial genus, and the horizontal axis indicates the correlation analysis with age, and three other clinical parameters. NIBUT: Non-invasive tear break up times (in seconds), redness: average temporal bulbar redness quantified automatically by Oculus Keratograph K5M; Schirmer: Schirmer I test results (mm over 5 min). The value of the Spearman correlation coefficient, which measures the strength and direction of association between two ranked variables, is color coded. Strong red color indicates positive correlation and strong blue color indicates negative or inverse correlation. Weak or faint color indicates weak or no correlation. **(A)** All participants included in analysis, and **(B)** Correlation stratified according to dry eye status (with or without dry eye). *p < 0.05; ^**^p < 0.01.

In univariate analyses, we found significant correlations between abundance of certain microbial genera and clinical parameters (Figure 3). For example, older age was correlated with a higher abundance of *Streptococcus* spp. (*p* < 0.01), and a lower abundance of *Staphylococcus* was correlated with a reduced Schirmer’s test reading (*p* < 0.01, First 2 rows Figure 3A), which indicates the presence of aqueous tear deficient dry eye.

Although *Staphylococcus* was not significantly correlated to age (second row Figure 3A), however, in older participants a few cases of *Firmicutes* (which included *Staphylococcus*) exceeded 10% relative abundance (Supplementary Figure 3). There is still a possibility that age may confound the relationship of *Staphylococcus* with Schirmer results, since it is well known that older age is weakly associated with reduced Schirmer readings (48, 52).

The fungal genus *Malassezia* also showed a similar, though less marked association with the Schirmer’s test result (*p* < 0.05, Figure 3A). Interestingly, when the same data were stratified by dry eye status, certain correlations remained in the dry eye group but not in the control group (Figure 3B). For example, the genus *Streptococcus* was highly correlated to age (*p* < 0.01), only in the dry eye group but not in the control group. On the other hand, the genus *Microbacterium* was increased in participants with more severe conjunctival redness (*p* < 0.01), but only in the control group, not for the dry eye participants.

**FIGURE 4:**
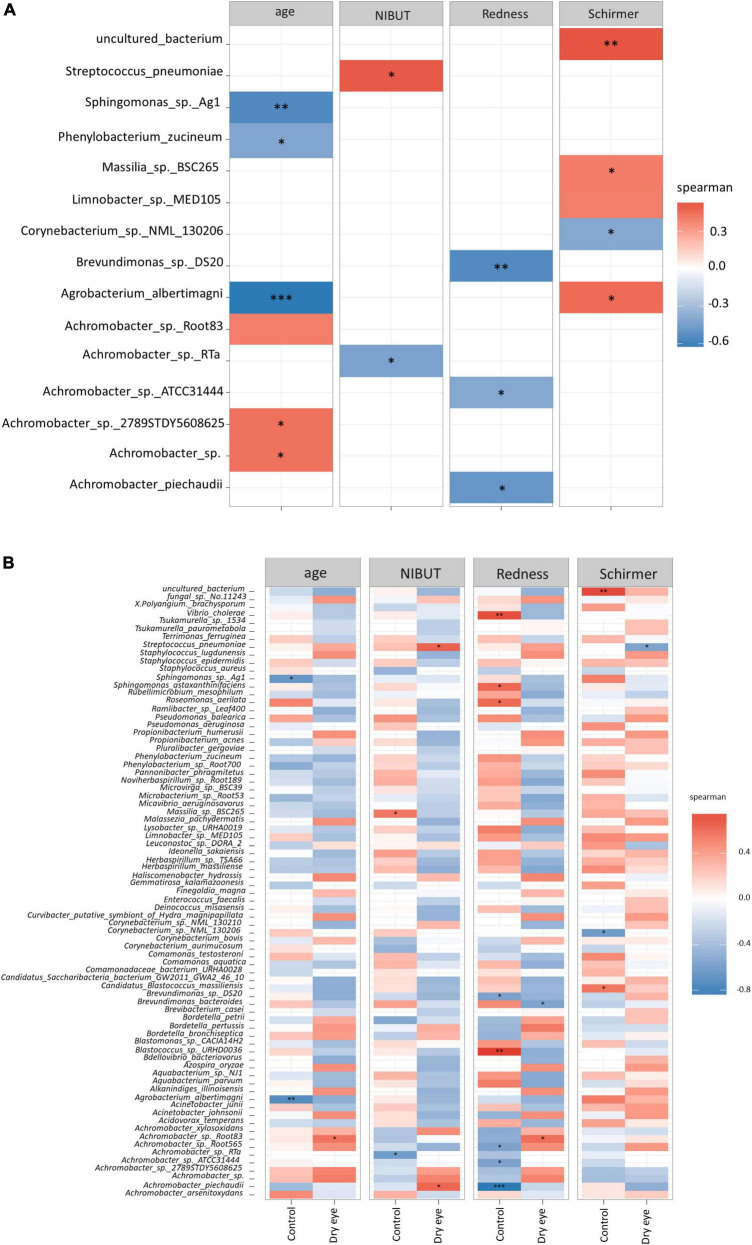
Correlation between microbial species and demographic/clinical parameters. Each horizontal row indicates a unique microbial species, and the horizontal axis indicates the correlation analysis with age, and three other clinical parameters. NIBUT: Non-invasive tear break up times (in seconds), Redness: Average temporal bulbar redness quantified automatically by Oculus Keratograph K5M; Schirmer: Schirmer I test results (mm over 5 min). The value of the Spearman correlation coefficient, which measures the strength and direction of association between two ranked variables, is color coded. Strong red color indicates positive correlation and strong blue color indicates negative or inverse correlation. Weak or faint color indicates weak or no correlation. **(A)** All participants included in analysis, and **(B)** Correlation stratified according to dry eye status (with or without dry eye). *p < 0.05; ^**^p < 0.01; ^***^p < 0.001.

At the species level, significant correlations were also observed (Figure 4) between the increased proportion of some species with older age and reduced proportion of other species with increased conjunctival redness (Figure 4A). When the results were stratified by dry eye status, some differential findings were observed between the control and dry eye groups (Figure 4B).

Next, we analyzed the abundance of functional genes not aligned with human genomes. The assessment of composition and abundance of functional genes can elucidate potential differences in microbial function. Such functional rather than taxonomic gene description can more accurately describe microbial community composition in specific host cohorts (53, 54). Our analysis revealed that in each of the samples, more than half the classified reads encode for genes related to metabolism, and of the remainder, about two thirds encoded for environmental/processing genes, while one third of the genes was associated with processing of genetic information. Approximately 20–30% of the assigned reads encoded for membrane transport, with 25% of these for ABC transporters (data not shown). Our analysis did not identify significant differences in the relative proportion of functional genes between the dry eye and control participants (data not shown).

**FIGURE 5:**
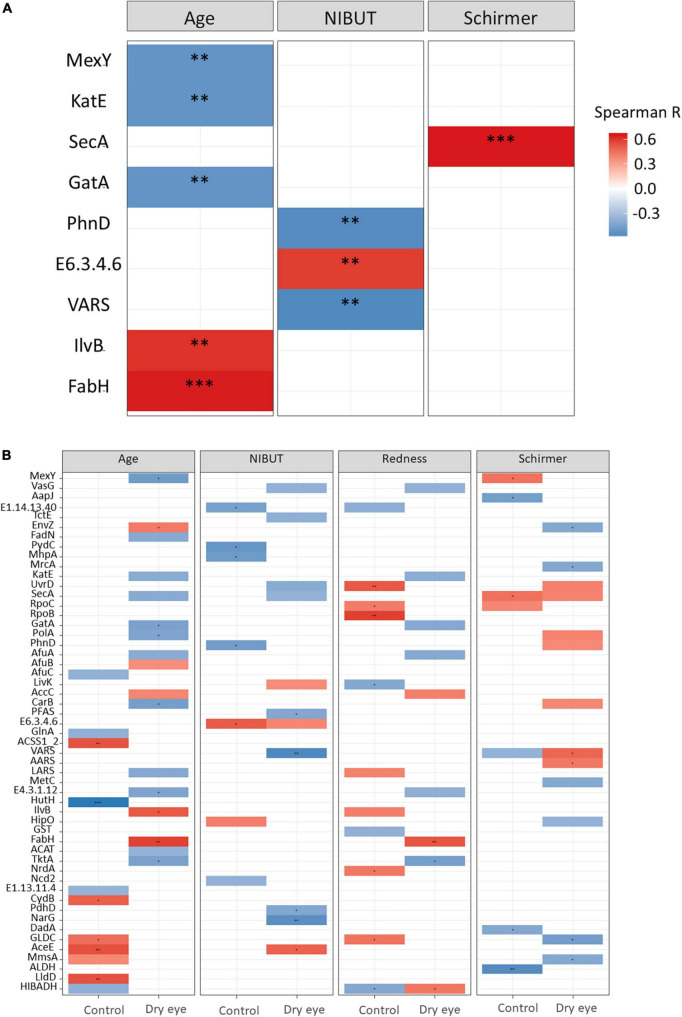
Results of functional gene analysis. **(A)** Correlation between KEGG functional classes and age, non-invasive tear break-up times (NIBUT) and Schirmer test for all participants. **(B)** Relationship between functional genes and age, NIBUT, conjunctival redness and Schirmer test, stratified by dry eye and control participants. The value of the Spearman correlation coefficient, which measures the strength and direction of association between two ranked variables, is color-coded. Red indicates positive correlation and blue indicates negative or inverse correlation. *p < 0.05; ^**^p < 0.01; ^***^p < 0.001. Supplementary Tables 4, 5 listed the full names of KEGG functional classes.

Correlating the functional classifications with clinical parameters revealed that glutathione S-transferase [EC:2.5.1.18] was higher in the controls than in dry eye individuals (*p* = 0.01). The preprotein translocase subunit SecA (K03070) was positively correlated to the Schirmer’s test result (*p* < 0.001, Figure 5A and Supplementary Table 4), while the 3-oxoacyl-[acyl-carrier-protein] synthase III protein [EC:2.3.1.180] (K00648) was positively correlated to age (*p* < 0.001, Figure 5A). The DNA-directed RNA polymerase subunit beta [EC:2.7.7.6] (K03043) was positively correlated to conjunctival redness (*p* < 0.01, Figure 5B and Supplementary Table 5) in control participants without dry eye.

### Achromobacter effects the cytokine levels of human cornea epithelial cells

As *Achromobacter* species are the predominant microorganisms, the potential functional significance of *Achromobacter* in human ocular surface physiology was examined. To achieve this, human corneal epithelial cells (HCE-T) were cultured with three strains of *A. xylosoxidans*, the most abundant species.

**FIGURE 6:**
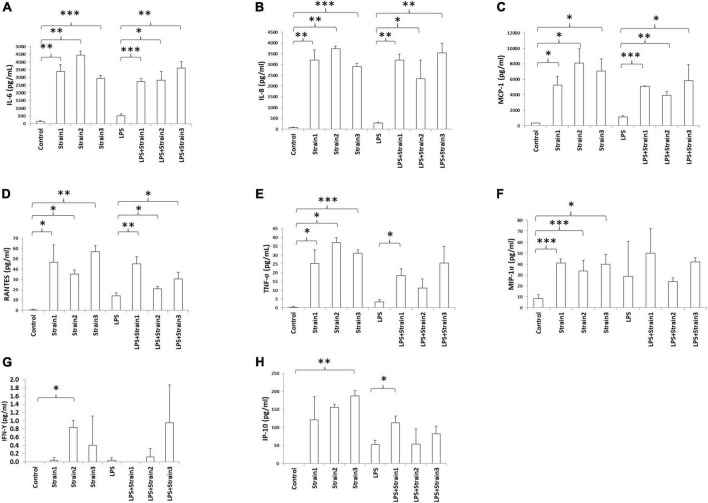
*Achromobacter* induced cytokine production in human corneal epithelial cells (serum free culture supernatant). **(A)** IL-6; **(B)** IL-8; **(C)** MCP-1; **(D)** RANTES; **(E)** TNF-α; **(F)** MIP-1α; **(G)** IFN-γ; **(H)** IP-10. IL, interleukin; TNF-α, tumor necrosis factor alpha; MIP, macrophage inhibitory protein; MCP, monocyte chemotactic protein; IFN, interferon; IP, interferon gamma-induced protein; RANTES, regulated on activation normal T cell expressed and secreted. The height of bars indicates the mean of three biological replicates. The error bars indicate one standard deviation *p < 0.05, ^**^p < 0.01, ^***^p < 0.001.

Addition of any of the three strains of *A. xylosoxidans* to the culture medium of HCE-T cells induced the upregulation of inflammatory cytokines, including IL-6, IL-8, MCP-1, RANTES, TNF-α, and MIP-1α (Figures 6A–F). Two strains of *A. xylosoxidans* upregulated IFN-γ (Figure 6G), while only one strain upregulated IP-10 (Figure 6H). When LPS was added to simulate ocular surface stress and the simultaneous presence of other gram-negative bacteria, all three strains of *A xylosoxidans* further upregulated IL-6, IL-8, MCP-1 and RANTES (Figures 6A–D). Only strain 1 further upregulated TNF-α and IP-10 (Figure 6).

**FIGURE 7:**
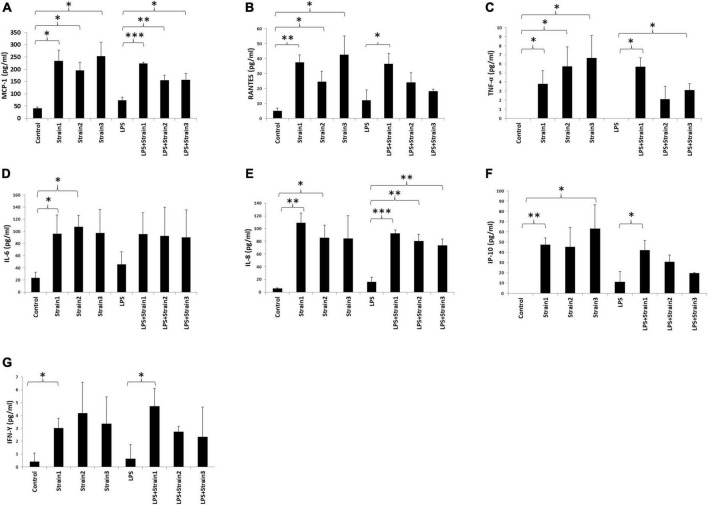
*Achromobacter* induced cytokine production in human corneal epithelial cells (cell lysates). **(A)** MCP-1; **(B)** RANTES; **(C)** TNF-*upalpha*; **(D)** IL-6; **(E)** IL-8; **(F)** IP-10; **(G)** IFN-γ. IL, interleukin; TNF, tumor necrosis factor; MIP, macrophage inhibitory protein; MCP, monocyte chemotactic protein; IFN, interferon; IP, interferon gamma-induced protein; RANTES, regulated on activation normal T cell expressed and secreted. The height of bars indicates the mean of three biological replicates. The error bars indicate one standard deviation *p < 0.05, ^**^p < 0.01, ^***^p < 0.001.

The three strains of *A. xylosoxidans*, when added to the medium of HCE-T cells induced upregulation of MCP-1, RANTES and TNF-α intracellularly (Figures 7A–C). Strains 1 and 2 of *A. xylosoxidans* upregulated intracellular IL-6 and IL-8 (Figures 7D,E). Strains 1 and 3 upregulated IP-10 whereas only strain 1 upregulated IFN-γ (Figures 7F,G). When LPS was added concurrently, all the strains of *A. xylosoxidans further* upregulated MCP-1 and IL-8 (Figures 7A,E). Strains 1 and 3 further upregulated TNF-α (Figure 7C), whereas strain 1 further upregulated RANTES, IP-10 and IFN-γ (Figures 7B,F,G).

Hence, addition of *A. xyloxidans* to corneal epithelial cells led to increased inflammatory cytokine production. In LPS-stimulated cells, cytokine production further increased when bacteria were present. An interesting finding was observed for the regulation of IP-10 and TNF-α by strain 2 and 3 of *A. xylosoxidans* (Figures 6, 7). Addition of LPS and *A. xylosoxidans* did not upregulate these cytokines to the same extent as adding only *A. xylosoxidans*. This suggests that LPS and these two strains of *A. xylosoxidans* may compete for the same cellular receptor in these epithelial cells.

## Discussion

Here we describe a comprehensive study of ocular surface microbiome in participants with mild type of dry eye using shotgun metagenomics. Studies based on 16S rRNA sequencing have indicated that dry eye participants may have a distinct ocular surface microbial community (Supplementary Table 6). This study characterized conjunctival microbiomes with *Achromobacter* being the numerically dominant bacterial genus. *A. xylosoxidans* may regulate production of cytokines in corneal epithelial cells. Age has a significant influence on the ocular surface microbiome, with older participants showing an increase in the proportion of bacterial genera such as *Achromobacter* and *Streptococcus*, but a reduced proportion of up to 23 bacterial species, mostly from the phylum *Proteobacteria.* Although there was no significant difference in the composition of conjunctival microbiomes in mild dry eye and control participants, the proportion of certain microbial genera was correlated to tear function, for example, a reduced abundance of *Staphylococcus* was correlated to decreased Schirmer’s test scores.

During the past 5 years, there was a dramatic increase in studies describing microbial communities with metagenomic analysis. We compared our work with similar studies by conducting literature research in the Medline database using “ocular surface microbiome” as keywords. Only studies performing either 16S rRNA or shotgun metagenomics on human subjects were included. Studies profiling microbial compositions of healthy ocular surfaces are summarized in Table 3. These studies revealed a more diverse microbial community on human ocular surfaces than traditional culture methods. Similar to other mucosal surfaces, the ocular surface microbiota comprises Gram-positive and Gram-negative bacteria, virus and fungi.

**TABLE 3:** Studies of healthy ocular surface microbiome.

Author, year	Location	Sample	Sequencing method	Species/Genus/Phylum of commensal
Kang et al. (37)	Wenzhou, China	Conjunctival swab	Shotgun metagenomic	Two phyla, 70 genera, and 140 species high relative abundances and positivity rates: *Streptococcus* pyogenes, *Staphylococcus epidermidis*, *Propionibacterium acnes, Corynebacterium accolens*, and *Enhydrobacter aerosaccus*.
Deng et al. (38)	Three cities, China	Conjunctival swab	Shotgun metagenomic	Predominant species*: Propionibacterium acnes* and *Staphylococcus, Epidermidis*, opportunistic pathogen *Micrococcus luteus* and *Staphylococcus haemolyticus* also found Phylum level: *Actinobacteria, Bacteroidetes, Chlamydiae, Chorobi, Deinococcus-thermus, Firmicutes, Fusobacteria, Proteobacteria, Spirochetes and Tenericutes*
Wen et al. (41)	Guangzhou, China	Conjunctival swab	Shotgun metagenomic	The most predominant species: *Propionibacterium acnes, Staphylococcus epidermidis, Escherichia coli, Micrococcus luteus, Ochrobactrum anthropic, Acidovorax* sp., *Acidovorax ebreus, Acinetobacter baumannii, Pseudomonas aeruginosa, Staphylococcus haemolyticus*
Dong et al. (34)	US	Conjunctival swab	16S rRNA	Core microbiota at genera level: *Pseudomonas, Propionibacterium, Bradyrhizobium, Corynebacterium, Acinetobacter, Brevundimonas, Staphylococci, Aquabacterium, Sphingomonas, Streptococcus, Streptophyta*, and *Methylobacterium*
Huang et al. (64)	Qingdao, China	Conjunctival swab	16S rRNA	The most predominant 10 phyla: P*roteobacteria, Actinobacteria, Firmicutes, Bacteroidetes, Deinococcuse-Thermus, Fusobacteria, Cyanobacteria/Chloroplast, Acidobacteria, Candidatus Saccharibacteria and Spirochetes.* The most predominant 10 genera: *Corynebacterium, Pseudomonas, Staphylococcus, Acinetobacter, Streptococcus, Millisia, Anaerococcus, Finegoldia, Simonsiella and Veillonella*
Doan et al. (65)	Seattle, USA	Conjunctival, buccal, cheek swabs	16S rRNA	Most predominant 4 genera: *Corynebacteria, Propionibacteria, Staphylococcus, and Streptococcus*
Ozkan et al. (66)	Australia	Conjunctival swabs, baseline, 1 and 3 months	16s rRNA	By culture, most predominant phyla: *Firmicutes, Actinobacteria and Proteobacteria*; most frequent genera: *Staphylococcus*, *Proprionibacterium*, *Micrococcus* and *Corynebacterium* By16s rRNA, most predominant phyla: *Proteobacteria*, *Firmicutes* and *Actinobacteria*; most frequent genera: *Corynebacterium*, *Acinetobacteria*, *Pseudomonas*, *Sphingomonas*, *Streptococcus*, *Massilia*, and *Rothia*
Ozkan et al. (67)	Australia	Conjunctival swabs (from pterygium surgery)	16S rRNA	*Pseudomonas* dominated the fornix and limbus. *Corynebacterium, Streptococcus*, and *Serratia* dominated in surface samples and low in the fornix and limbus samples. *Acinetobacter* and *Thermoanerobacterium* similar among groups
Cavuoto et al. (56)	USA	Conjunctival, lid margin swabs	16S rRNA	Phylum level*: Proteobacteria Firmicutes, Bacteroidetes* and *Actinobacteria* abundant in both children and adult; *Proteobacteria, Fusobacteria, Firmicutes*, and *Bacteroidetes* depleted in adults, while *Actinobacteria* increased. Genus level: *Streptococcus, Staphylococcus*, and *Brachybacterium* reduced, while *Corynebacterium, Paracoccus*, and *Propionibacterium* increased.
Cavuoto et al. (55)	USA	Conjunctival nasal, throat swabs	16S rRNA	Most abundant phyla: *Firmicutes, Proteobacteria, Actinobacteria, Cyanobacteria*, and *Bacteroidetes.* Most abundant family: *Staphylococcaceae, Streptococcaceae, Corynebacteriaceae, Moraxellaceae, Enterobacteriaceae, Oceanospirillaceae, and Bacillaceae*. *Staphylococcus* species predominant
Cavuoto et al. (68)	US	Conjunctival swab, eyelid margin, periocular skin	16S rRNA	*Proteobacteria, Bacteroidetes* dominated eyelid margin, whereas *Firmicutes* dominated periocular skin.
Fan et al. (57)	Qingdao, China	Conjunctival swab	16S rRNA	*Corynebacterium, Pseudomonas, Staphylococcus, Acinetobacter and Streptococcus* dominated before treatment. After treatment with 5.0% PVI, *Pseudomonas, Corynebacterium* and *Acinetobacter* predominant
Suzuki et al. (69)	Japan	Eyelid, meibum, conjunctival sac, lower-eyelid skin	16s rRNA	*P. acnes* or P*seudomonas* sp. dominated meibum; *P. acnes* for conjunctival sac. *Corynebacterium* sp. or the *Neisseriaceae* dominant in elderly.
Matysiak et al. (62)	Poland	cornea tissues; conjunctival swab	RNA-seq, traditional culture and PCR	By conventional and molecular methods, most dominant phylum in ocular samples: *Proteobacteria, Firmicutes* and *Actinobateria.*
Ozkan et al. (70)	Australia	Eyelid margin tissues; fornix and limbus conjunctival tissues, conjunctival, facial skin swabs	16S rRNA	*Corynebacterium, Staphylococcus* resident on skin and lid margin; *Corynebacterium, Staphylococcus* mainly on ocular surface; *Pseudomonas* mainly on conjunctival and lid margin.

Our finding that age is the primary factor affecting the conjunctival microbiome is in agreement with previous studies. In a study investigating ocular surface microbiome of healthy individuals, old and young participants were clearly separated in PCA analysis. Compared to the younger group, the older cohort had significantly greater abundance of several *Streptococcus* species, and also altered carbohydrate, lipid, nucleotide and amino metabolic pathways (41). Children above 6 months old had a higher abundance of *Proteobacteria* and reduced *Firmicutes* than toddlers (<6 months old) (55). Compared to adults, there was an increase of *Streptococcus* and *Staphylococcus* OTUs in children below 8 years old (56).

Recently, a study compared conjunctival microbiome compositions of healthy subjects from the three cities Beijing, Wenzhou and Guangzhou, which have distinct climates and diets (38). Shotgun metagenomic sequencing revealed that the conjunctival microbiome of Beijing participants showed distinct characteristics compared to Guangzhou and Wenzhou microbiomes, while there was no significant difference between Guangzhou and Wenzhou participants, suggesting that the environment shapes their conjunctival microbiota. Furthermore, for the participants who have traveled to a different city for at least 15 days, the conjunctival microbiome was markedly changed (38). These findings strongly supported an environmental impact on the composition of conjunctival microbiome. In our study, *Achromobacter* was the most abundant genus of the conjunctival microbiomes of all subjects. This finding was different from the outcome of the study by Deng et al. (38) possibly due to the different climate and diet in Singapore. In another study conducted in a coastal city from China, *Achromobacter* was one of the nine abundant genera in all patients tested (57).

In closed eye tears, the microbial ecology (determined by 16S sequencing) of tear samples from normal participants and from patients with mild dry eye showed no significant difference, but clear differences were observed between participants with mild and moderate dry eye (2). Therefore, that study supported our findings that our mild dry eye participants showed no alteration of microbiome from normal participants. If the mild dry eye cases don’t have a different microbiome from controls, it suggests that until more severe dry eye is reached, the level of immunoregulation on the ocular surface may not be drastically perturbed.

*A. xylosoxidans* is a Gram-negative aerobic, oxidase- and catalase-positive, motile bacterium with peritrichous flagella found in unsanitary conditions, soil and water. While the exact function of *Achromobacter* spp. in the normal eye is not known, they are likely commensals. In dry eye disease, it was one of the more variable genera (2). In fungal keratitis, it was also one of the most abundant genera detected (11). We found several proinflammatory cytokines to be upregulated when cultured human corneal epithelial cells were exposed to the three strains of this bacterium tested here, with the morphology of the cells remaining normal. Some of these dysregulated cytokines have been reported to be elevated in the tear fluid of people with dry eye (58). The TLR ligand LPS is the most abundant cell wall component of Gram-negative bacteria, including those of *Achromobacter*. LPS from different bacteria may compete for the same cellular receptors, so partially inhibitory relationships may exist between different Gram-negative bacteria.

Our hypothesis is that with increasing age, there is an altered immunoregulatory influence due to the change in composition of the microbiome. There is an increase in *Achromobacter* spp., and reduction in several other species of bacteria and fungi. Because of the change in the microbial ecosystem, there is reduced tolerance and increased prevalence of inflammatory conditions, such as dry eye. In fact, the increase in CD4^+^ T lymphocytes in the conjunctiva of older healthy people (59) is consistent with this concept. Some of the bacteria found to be reduced in the elderly, such as *Micavibrio aeruginosavorus*, normally feeds on pathogenic *Pseudomonas aeruginosa* (60). Hence, this finding may explain the increased susceptibility to ocular surface inflammation and infection in old age. A few redundant members of the bacterial microbiome can serve to maintain functionality of the community (61). Reduction of some of the 23 species listed in Supplementary Table 3 may not impact on conjunctival mucosal defense. Supplementary Tables 6, 7 summarize the studies of conjunctival microbiomes in dry eye and other ocular surface diseases, respectively.

Our study employed shotgun whole genome sequencing metagenomic analysis for characterizing the ocular microbiome. All participants for the eye microbiome investigation were subjected to standard characterization, including objective measurements of tear break up times. One of the limitations of the study is that we only examined superficial conjunctival fornix, and the results may not be applicable to microbiomes of the cornea or the bulbar conjunctiva. A study has shown that cornea and conjunctival microbiota are different (62). In addition, our experiments with *Achromobacter* were entirely *in vitro*, and it would be beneficial to evaluate the response induced by *Achromobacter* spp. inoculation on the ocular surface in animal disease models. We only tested the effect of *Achromobacter* spp. on human corneal epithelial cells as it was difficult to get conjunctival epithelial cells. We did not investigate whether the above effects on the cultured cells are specific to *Achromobacter.* Since the microbiome composition may be influenced by environmental and occupational factors, it may not be possible to extrapolate the results to participants from a different setting. It is not possible to delineate whether it is the external climate or the indoor conditions that shape this microbiome, though the China study that evaluated three cities suggest inter-center variability more than intra-center findings. Our sample size was small, it is possible that statistically significant differences may be revealed by larger sample sizes, but in the literature, similar sample sizes were able to detect changes in severe MGD (33, 63). Tetracaine was applied before sample collection. However, the possibility of tetracaine to introduce contaminant DNA is very low, since it was instilled from sterile unit dose (single use) vials which were discarded after application by each participant. Tetracaine was used for both the dry eye and comparison participants groups. Tetracaine may reduce diversity, but we believe the effect of tetracaine to diversity is limited.

## Conclusion

In conclusion, we report the results derived from a comprehensive characterization of the ocular surface microbiome in participants with mild dry eye and control individuals. In the normal ocular microbiome, the phylum *Proteobacteria* dominates, with presence of *Achromobacter* spp. which increases in abundance with age. We also demonstrated that strains of *A. xylosoxidans* induced cytokine expression in basal and stressed epithelial cells. The alteration of the ocular surface microbial ecosystem with age may influence its susceptibility to inflammation.

## Data availability statement

The data presented in this study are deposited in the NCBI Sequence Read Archive repository, accession number: PRJNA886972.

## Ethics statement

The studies involving human participants were reviewed and approved by the SingHealth Centralized Institutional Review Board. The patients/participants provided their written informed consent to participate in this study.

## Author contributions

LT and SK were the principle investigators of the study, provided study funding, participated in the study design, data interpretation, and wrote the manuscript. LT performed clinical assessment of the participants. LY, DD-M, and SS contributed to the preparation and acquisition of sequencing data. FC and RW participated in the bioinformatics analysis and the revision of the manuscript. AH and SC contributed to the acquisition of the *Achromobacter* treatment data and revision of the manuscript. All authors read and approved the final manuscript and contributed to the article and approved the submitted version.

## Footnotes

1
ftp.ncbi.nlm.nih.gov/blast/db/FASTA/nr.gz
