# Supplementary material for: Shotgun metagenomic sequencing analysis of ocular surface microbiome in Singapore residents with mild dry eye
Source: Front Med (Lausanne). 2022 Nov 10;9:1034131. doi: 10.3389/fmed.2022.1034131 (PMC9684611; doi:10.3389/fmed.2022.1034131)
Supplement: Supplementary file 1 [file Data_Sheet_1.docx]

**Table S1. Clinical features of participants**^+^

| **Patient ID** | **Schirmer R** | **Schirmer L** | **NIBUT R** | **NIBUT L** | **Type of Dry Eye #** |
| --- | --- | --- | --- | --- | --- |
| MB_D012 | 17 | 22 | 5.2 | 2.9 | EDE |
| MB_D014 | 12 | 25 | 7.7 | 8.1 | EDE |
| MB_D015 | 22 | 15 | 5.7 | 9.9 | EDE |
| MB_D016 | 18 | 19 | 7.3 | 13.3 | EDE |
| MB_D018 | 6 | 7 | MTS | 7.8 | MDE |
| MB_D019 | 19 | 12 | 7.7 | 6.4 | EDE |
| MB_D020 | 30 | 14 | 8.8 | 5.5 | EDE |
| MB_D021 | 29 | 19 | 5.9 | 5.2 | EDE |
| MB_D022 | 10 | 7 | 4.4 | 18.2 | MDE |
| MB_D024 | 3 | 1 | 5.9 | 12.9 | MDE |
| MB_D030 | 22 | 15 | 24.9 | 7.1 | MDE |
| MB_D032 | 17 | 22 | 6.9 | 2.5 | EDE |
| MB_D036 | 4 | 4 | 11.5 | 10.9 | ADDE |
| MB_D037 | 9 | 11 | 13.6 | 24.1 | ++ |

#

EDE : evaporative dry eye (Schirmer I>7mm, NIBUT<10s),

MDE : mixed dry eye (Schirmer I<=7 mm, NIBUT<10s),

ADDE : aqueous deficient dry eye (Schirmer I<=7mm, NIBUT>10s)

When the NIBUT is too short to register, it will be recorded as 0 s.

+ None of the patients have central corneal staining. Some have mild staining in one zone either nasal, temporal, inferior or superior zone.

++ This patient could not be easily classified into dry eye subtype because the patient has been treated with refresh tears which could have altered the dry eye parameters. It is also possible the patient has mucin deficiency subtype but this was not confirmed.

**Table S2 Artificial tears and contact lens wear of dry eye participants**

| **Patient ID** | **Previous Eyedrop** | **Contact lens** |
| --- | --- | --- |
| MB_D018 | Refresh | Daily, 12hrs/day, 6days/week |
| MB_D015 | Refresh Plus | none^*^ |
| MB_D019 | Tears naturale free | none^*^ |
| MB_D020 | Refresh | none^*^ |
| MB_D030 | Eyemo | none^*^ |
| MB_D036 | Systane ultra | none^*^ |
| MB_D037 | Refresh | none^*^ |
| MB_D022 | none^*^ | Daily, 2x/month, 3hrs/day |
| MB_D012 | none^*^ | Fortnightly, 12hrs/day, 5days/week |
| MB_D016 | none^*^ | Monthly, 12hrs/day, 7x/week |
| MB_D021 | none^*^ | Monthly, 10hrs/day, 4/week |
| MB_D014 | none^*^ | none^*^ |
| MB_D024 | none^*^ | none^*^ |
| MB_D032 | none^*^ | none^*^ |

*No known

**Table S3. Bacterial species under-represented in the smaller cluster of older individuals**

| **Specie** | **Phylum/Class** | **Morphology** | **Characteristics** |
| --- | --- | --- | --- |
| *Vibrio cholerae* | Gammaproteobacteria | Gram negative | Motile Facultative anaerobe  Intestinal infection |
| *Pannonibacter phragmitetus* | Alphaproteobacteria | Gram negative | Cause liver abscess |
| *Microbacterium sp Root53* | Actinobacteria | Gram positive rods | Found in clinical specimens and in soil |
| *Lysobacter sp URHA0019* | Gammaproteobacteria | Gram negative bacillus | Motile, antimicrobial effects on bacteria |
| *Blastomonas sp CACIA14H2* | Alpha4 proteobacteria | Gram negative bacillus | Aerobic, from soil and water |
| *Sphingomonas astaxanthinifaciens* | Alphaproteobacteria | Gram negative bacillus | Aerobic |
| *Sphingomonas sp Ag1* | Alphaproteobacteria | Gram negative bacillus | Aerobic |
| *Massilia sp BSC265* | Betaproteobacteria | Gram negative bacillus | Motile, found in soil |
| *Deinococcus misasensis* | Deinococcus-Thermus | Deinoxanthin and thick cell wall | Survives under gamma radiation |
| *Acidovorax temperans* | Betaproteobacteria | Gram negative | Potential rice pathogen |
| *Phenylobacterium zucineum* | Alphaproteobacteria | Gram negative rod | Facultative intracellular bacterium |
| *Phenylobacterium sp Root700* | Alphaproteobacteria | Gram negative rod | Plant pathogen |
| *Noviherbaspirillum sp Root189* | Betaproteobacteria | Gram negative | Soil bacteria |
| *Herbaspirillum massiliense* | Betaproteobacteria | Gram negative | Soil bacteria |
| *Herbaspirillum sp TSA66* | Betaproteobacteria | Gram negative | Soil bacteria |
| *Rubellimicrobium mesophilum* | Roseobacter | Pigmented | Soil bacteria |
| *Comamonas aquatica* | Betaproteobacteria | Gram negative | Motile bacteria |
| *Aquabacterium parvum* | Betaproteobacteria | Gram negative  bacillus | Found in biofilms of drinking water |
| *Aquabacterium sp NJ1* | Betaproteobacteria | Gram negative  bacillus | NA |
| *Candidatus Blastococcus massiliensis* | Actinobacteria | Gram positive | NA |
| *Blastococcus_sp._URHD0036* | Actinabacteria | Gram positive | NA |
| *Ideonella sakaiensis* | Betaproteobacteria | Gram negative | Breaks down PET plastic |
| *Micavibrio aeruginosavorus* | Alphaproteobacteria | Gram negative | Obligate Epibiotic bacteria predator, Preys on *Pseudomonas aeruginosa* bacteria |

NA: not available

**Table S4 Full names of the KEGG functional classes for Figure 5A**

| **Entry** | **Name** | **Definition** |
| --- | --- | --- |
| K18095 | mexY, amrB | multidrug efflux pump |
| K03781 | katE, CAT, catB, srpA | catalase |
| K03070 | SecA | preprotein translocase subunit SecA |
| K02433 | gatA, QRSL1 | aspartyl-tRNA(Asn)/glutamyl-tRNA(Gln) amidotransferase subunit A |
| K02044 | phnD | phosphonate transport system substrate-binding protein |
| K01941 | E6.3.4.6 | urea carboxylase |
| K01873 | VARS, valS | valyl-tRNA synthetase |
| K01652 | E2.2.1.6L, ilvB, ilvG, ilvI | acetolactate synthase I/II/III large subunit |
| K00648 | fabH | 3-oxoacyl-[acyl-carrier-protein] synthase III |

**Table S5 Full names of the KEGG functional classes for Figure 5B**

| **Entry** | **Name** | **Definition** |
| --- | --- | --- |
| K18095 | mexY, amrB | multidrug efflux pump |
| K11907 | vasG, clpV | type VI secretion system protein VasG |
| K09969 | aapJ, bztA | general L-amino acid transport system substrate-binding protein |
| K09461 | E1.14.13.40 | anthraniloyl-CoA monooxygenase |
| K07649 | tctE | two-component system, OmpR family, sensor histidine kinase TctE |
| K07638 | envZ | two-component system, OmpR family, osmolarity sensor histidine kinase EnvZ |
| K07516 | fadN | 3-hydroxyacyl-CoA dehydrogenase |
| K06016 | pydC | beta-ureidopropionase / N-carbamoyl-L-amino-acid hydrolase |
| K05712 | mhpA | 3-(3-hydroxy-phenyl)propionate hydroxylase |
| K05366 | mrcA | penicillin-binding protein 1A |
| K03781 | katE, CAT, catB, srpA | catalase |
| K03657 | uvrD, pcrA | DNA helicase II / ATP-dependent DNA helicase PcrA |
| K03070 | SecA | preprotein translocase subunit SecA |
| K03046 | rpoC | DNA-directed RNA polymerase subunit beta' |
| K03043 | rpoB | DNA-directed RNA polymerase subunit beta |
| K02433 | gatA, QRSL1 | aspartyl-tRNA(Asn)/glutamyl-tRNA(Gln) amidotransferase subunit A |
| K02335 | polA | DNA polymerase I |
| K02044 | phnD | phosphonate transport system substrate-binding protein |
| K02012 | afuA, fbpA | iron(III) transport system substrate-binding protein |
| K02011 | afuB, fbpB | iron(III) transport system permease protein |
| K02010 | afuC, fbpC | iron(III) transport system ATP-binding protein |
| K01999 | livK | branched-chain amino acid transport system substrate-binding protein |
| K01961 | accC | acetyl-CoA carboxylase, biotin carboxylase subunit |
| K01955 | carB, CPA2 | carbamoyl-phosphate synthase large subunit |
| K01952 | PFAS, purL | phosphoribosylformylglycinamidine synthase |
| K01941 | E6.3.4.6 | urea carboxylase |
| K01915 | glnA, GLUL | glutamine synthetase |
| K01895 | ACSS1_2, acs | acetyl-CoA synthetase |
| K01873 | VARS, valS | valyl-tRNA synthetase |
| K01872 | AARS, alaS | alanyl-tRNA synthetase |
| K01869 | LARS, leuS | leucyl-tRNA synthetase |
| K01760 | metC | cysteine-S-conjugate beta-lyase |
| K01750 | E4.3.1.12, ocd | ornithine cyclodeaminase |
| K01745 | hutH, HAL | histidine ammonia-lyase |
| K01652 | E2.2.1.6L, ilvB, ilvG, ilvI | acetolactate synthase I/II/III large subunit |
| K01451 | hipO | hippurate hydrolase |
| K00799 | GST, gst | glutathione S-transferase |
| K00648 | fabH | 3-oxoacyl-[acyl-carrier-protein] synthase III |
| K00626 | ACAT, atoB | acetyl-CoA C-acetyltransferase |
| K00615 | E2.2.1.1, tktA, tktB | transketolase |
| K00525 | E1.17.4.1A, nrdA, nrdE | ribonucleoside-diphosphate reductase alpha chain |
| K00459 | ncd2, npd | nitronate monooxygenase |
| K00450 | E1.13.11.4 | gentisate 1,2-dioxygenase |
| K00426 | cydB | cytochrome bd ubiquinol oxidase subunit II |
| K00382 | DLD, lpd, pdhD | dihydrolipoamide dehydrogenase |
| K00370 | narG, narZ, nxrA | nitrate reductase / nitrite oxidoreductase, alpha subunit |
| K00285 | dadA | D-amino-acid dehydrogenase |
| K00281 | GLDC, gcvP | glycine dehydrogenase |
| K00163 | aceE | pyruvate dehydrogenase E1 component |
| K00140 | mmsA, iolA, ALDH6A1 | alonate-semialdehyde dehydrogenase (acetylating) / methylmalonate-semialdehyde dehydrogenase |
| K00128 | ALDH | aldehyde dehydrogenase (NAD+) |
| K00101 | lldD | L-lactate dehydrogenase (cytochrome) |
| K00020 | HIBADH, mmsB | 3-hydroxyisobutyrate dehydrogenase |

**Table S6-I Microbiome studies of Dry eye.**

| **Author, year** | **Disease** | **Location** | **Sample** | **Sequencing method** | **Species/genus/phylum of commensal** | **Immune/molecular alterations** | **Sample Size** |
| --- | --- | --- | --- | --- | --- | --- | --- |
| Andersson et al, 2021^1^ | aqueous tear-deficient DED | Denmark | Conjunctival swab | 16S rRNA | *Enhydrobacter, Brevibacterium, Staphylococcus, Streptococcus and Cutibacterium.* Biomarker for controls*: Pseudomonas*; Biomarkers for patients with aqueous tear-deficient dry eye: *Bacilli* | Decreased diversity and reduced relative abundances in DED. A minimal core ocular surface microbiota may exist. | Dry Eye: 39  Control: 28 |
| Kent Willis et al, 2020 ^2^ | DED | USA | Flush tears | 16S rRNA | Important identifiers of dry eye: *Methylobacterium, Megasphaera, Parabacteroides, S247, Bifidobacterium, Streptococcus, Desulfovibrio and Acetobacter* | DED microbial communities more diverse, distinct from the healthy, remains distinct despite daily saline eye wash | Dry Eye: 36  Normal Eye: 36 |
| Li Zhenhao et al, 2019 ^3^ | DED | Guang Zhou, China | Conjunctival swab | 16S rRNA | Top 10 (all samples): *Proteobacteria, Firmicutes, Bacteroidetes, Actino- bacteria, Cyanobacteria, Acido- bacteria, Chloroflexi, Plancto- mycetes, Epsilonbacteraeota, Verrucomicrobia*  DE: *Pseudomonas, Acinetobacter, Bacillus, Chryseobacterium, and Corynebacterium* | *Bacteroidia* and *Bacteroidetes* enriched in DE; *Pseudomonas* enriched in NDE. *Bacilli* dominated in MGD; *Bacteroidetes* dominated in NMGD among DE subjects. | Dry Eye: 35  Non Dry Eye: 54 |
| Graham et al., 2007^4^ | DED | UK | Conjunctival swabs | 16S rRNA | *S. epidermidis, Staphylococcus sp., uncultured bacterium, R. erythropolis, Propionibacterium, Corynebacterium sp., Erwinia sp., Klebsiella oxytoca, and Klebsiella sp. Bacillus sp., Propionbacterium acnes,* and *K. oxytoca* only in dry eye samples | Trend of increasing bacterial count with a decrease in goblet cells | DED: 34  Normal: 57 |

**Table S6-II Microbiome studies of Dry eye subtype**

| **Author, year** | **Disease** | **Location** | **Sample** | **Sequencing method** | **Species/genus/phylum of commensal** | **Immune/molecular alterations** |
| --- | --- | --- | --- | --- | --- | --- |
| Cintia S. de Paiva et al, 2016^5^ | SS | USA (TX) | Conjunctival inferior swab, tongue, stool | 16s rRNA | Phyla *Firmicutes, Actinobacteria, Proteobacteria* and *Bacteroidetes* dominated conjunctival samples from control, rosacea, and SS patients | SS marked by a dysbiotic intestinal microbiome |
| Zilliox Michael J et al, 2020 ^6^ | SJS, oGVHD, Floppy eyelid synd | USA (IL) | Conjunctival swab | 16s rRNA | *Actinomyces, Streptococcus, Rothia, Prevotella* and *Corynebacterium* from OSD patients. *Staphylococcus* predominant in SJS, *Corynebacterium* predominant in dry eye and lax eyelid syndrome. | Healthy eyes had a *Lactobacillus/Streptococcus* mix or *Corynebacterium* microbiome. *Staphylococcus* predominated in SJS, *Lactobacillus* in oGVHD, and *Corynebacterium* in DED and LES. |
| Thanachaporn Kittipibul et al, 2020 ^7^ | SJS | Thailand | Conjunctival swab | 16S rRNA | *Acrobacter, Streptococcus, Lactobacillus, Bacillus, Bifidobacterium, Bacteroides, Pseudomonas, Acinetobacter, Staphylococcus, Pseudoalteromonas, Clostridium* different between SJS and healthy | Ocular surface of SJS occupied by more diverse microorganisms with increased proportion of pathogenic species |
| Samantha Sagaser et al, 2021^8^ | Rosacea with dry eye symptoms | USA | Conjunctival swab | 16s rRNA | *Clostridium, Klebsiella, Brevibacterium, Lactobacillus, Neisseria, Streptococcus, Corynebacterium, Butyricicoccus*, and *Actinomyces* reduced after IPL-MGX or MGX | IPL treatment offered no additional benefit to MGX in decreasing virulent bacteria present on the ocular surface and not influence TGF-β levels in tears. |
| Yun Qi, et al, 2021 ^9^ | Autoimmune dry eye vs dry eye | China | Conjunctival swab | 16s rRNA | Phyla *Actinobacteria, Firmicutes,* and *Bacteroidetes* specific for immdry eye*; Proteobacteria* for the dry eye*.* Genus *Corynebacterium* biomarker for the immdry eye and *Pelomonas* for the dry eye. | Composition and function of ocular microbiome between subjects with autoimmune dry eye and dry eye were different. |

**Table S7-I Microbiome studies in other ocular surface disease: Inflammation and MGD**

| **Author, year** | **Disease** | **Location** | **Sample** | **Sequencing method** | **Species/genus/phylum of commensal** | **Immune/molecular alterations** |
| --- | --- | --- | --- | --- | --- | --- |
| Jiang et al, 2018^10^ | MGD | Beijing, China | Conjunctival swab, meibomian gland secretions | 16S rRNA | *S. epidermidis* frequently isolated in MG, no bacteria isolated from 80% of Conjunctival sac samples. *Staphylococcus,* *Corynebacterium* and *Microbacteriaceae* common between MG and conj sac. *Bacillus, Paenibacillus* and *Lysinibacillus* only detected in MG. The rate of *C. macginleyi* higher in severe MGD | *Corynebacterium macginleyi* only detected in the severe MGD group |
| Dong Xiaojin et al, 2019^11^ | MGD | Qingdao, China | Human, conj swab | 16S rRNA | Phylum level: *Actinobacteria, Firmicutes, Proteobacteria, Bacteroidetes and Deinococcus*.  Genus level: *Staphylococcus, Corynebacterium, Propionibacterium, Sphingomonas, Snodgrassella* and *Streptococcus.*  Species level, *Staphylococcus epidermidis, Staphylococcus aureus*. | *Staphylococcus, Corynebacterium*, and *Sphingomonas* may play roles in the pathophysiology of MGD. |
| Zhao et al., 2020^12^ | MGD | Zhejiang, China | Human, MGD patients vs controls | Shotgun metagenomic analysis | MGD meibum abundant of *Campylobacter coli*, *Campylobacter jejuni*, and *Enterococcus faecium* pathogens. | MGD meibum contains distinct microbiota with stronger immune evasive virulence. |

**Table S7-II Microbiome studies in other ocular surface disease: Inflammation**

| **Author, year** | **Disease** | **Location** | **Sample** | **Sequencing method** | **Species/genus/phylum of commensal** | **Immune/molecular alterations** |
| --- | --- | --- | --- | --- | --- | --- |
| Lee et al., 2012^13^ | Blepharitis | Korea | Conjunctival swabs, eyelash and tear samples | 16S rRNA sequencing | Common genera among all samples: *Actinobacteria Proteobacteria, Firmicutes, Cyanobacteria*, or *Bacteroidetes.* *Propionibacterium, Staphylococcus, Streptophyta, Corynebacterium*, and *Enhydrobacter* | Increased *Staphylococcus, Streptophyta, Corynebacterium*, and *Enhydrobacter*, decreased *Propionibacterium* in blepharitis subjects |
| Yan Y et al, 2020^14^ | demodex Blepharitis | Shanghai, China | Conjunctival swabs | 16S rRNA | *Firmicutes* and *Corynebacterium*, *Lactobacillus* and *Bifidobacterium* higher in patients. *Staphylococcus epidermidis* positively correlated with the demodex | Microbiota imbalance in patients with demodex blepharitis |
| Chao C. et al, 2018^15^ | soft contact lens (SCL) wearers +/- infiltrative events (CIEs) | USA | Conjunctival swabs | 16S rRNA | Predominant organisms: *Staphylococcus, Propionibacterium, Streptococcus,* and *Corynebacterium*. *Neisseria* genus higher in cases group | Cytokine concentration of IL-6 may related to susceptibility to CIE |
| Shin et al., 2016^16^ | Contact lens | USA | Conjunctival swabs, skin under eye, contact lens | 16S rRNA | *Pseudomonas, Acinetobacter, Methylobacterium*, and *Lactobacillus*. Decreased: *Haemophilus, Streptococcus, Staphylococcus,* and *Corynebacterium* | Higher *Haemophilus, Neisseria, Streptococcus, Staphylococcus, Rothia,* and *Corynebacterium* compared to skin |

**Table S7-III Microbiome studies in other ocular surface disease: Allergy**

| **Author, year** | **Disease** | **Location** | **Sample** | **Sequencing method** | **Species/genus/phylum of commensal** | **Immune/molecular alterations** |
| --- | --- | --- | --- | --- | --- | --- |
| Yau Jennifer et al, 2019^17^ | allergic rhinoconjunctivitis | Hong Kong | ocular and nasopharyngeal swabs | 16s rRNA | Phyla *Proteobacteria, Firmicute, Actinobacteris* abundant in all samples. | The alpha diversity of microbiome in ocular higher than nasopharyngeal |
| Liang Qiaoxing et al, 2021 ^18^ | Healthy control patients with PAC,  SAC and VKC | Guang zhou, China | Conjunctival swabs | Shotgun metagenomic | Identified genus*: Corynebacterium, Streptococcus, Herminimonas, Mycoplasma, Rothia, Staphylococcus, Lactobacillus, Malassezia* | *Brevibacterium aurantiacum* and *Staphylococcus sci*  *Uri, Streptococcus* species enriched in VKC. |
| Pratima Vishwakarma et al 2021^19^ | VKC vs control | India | Conjunctival swabs | 16s rRNA | Mainly *Staphylococcus* species identified | *Staphylococcus* predominant. |

**Table S7-IV Microbiome studies in other ocular surface disease: infection**

| **Author, year** | **Disease** | **Location** | **Sample** | **Sequencing method** | **Species/genus/phylum of commensal** |
| --- | --- | --- | --- | --- | --- |
| Butcher et al 2017^20^ | *Trachoma* | Solomon Islands | Conjunctiva swabs Children 1-9 years old | 16S rRNA | *Corynebacterium, Propionibacterium and Helicobacter.*  *In controls Corynebacterium, Paracoccus, Propionibacterium, and Neisseria* |
| Pickering et al., 2019^21^ | *Trachoma* | UK | Conjunctival swabs | 16S rRNA | Reduced ocular bacterial diversity in adults with scarring trachoma compared to controls, with increased relative abundance of *Corynebacterium* |
| Sisinthy Shivaji et al 2021^22^ | bacterial keratitis | India | Conjunctival swabs, Corneal scraps | 16S rRNA | Unable to obtain details |
| Cavuoto et al., 2021^23^ | unilateral keratitis and healthy controls | US | Conjunctival swab | 16S rRNA | Phyla *Proteobacteria, Actinobacteria*, and *Firmicutes* more than 10-fold greater, *Micrococcus* 100-fold higher, and *Cutibacterium* 10 fold greater, whereas *Proteobacteria, Ralstonia* 5.7 fold lower in patients |
| Kang, Y. et al, 2020^24^ | Traumatic Corneal ulcers | Wenzhou,  China | upper and  lower palpebral, caruncle, and conjunctival fornix swab | Shotgun metagenomic | *Proteobacteria, Actinobacteria,* and *Firmicutes*. *Pseudomonas, Streptococcus, Corynebacterium, Cronobacter* and *Staphylococcus* |
| Ge C. et al 2019 ^25^ | Fungal keratitis | Qingdao, China | corneal scrapes, conjunctival swab | 16S rRNA | *Pseudomonas, Sphingomona, Acinetobacter, Caulobacter, Achromobacter, Thermus and Rhodopseudomonas* dominant the conjunctival sac of the diseased eye. |

**Table S7-V Microbiome studies in other ocular surface disease: diabetes**

| **Author, year** | **Disease** | **Location** | **Sample** | **Sequencing method** | **Species/genus/phylum of commensal** |
| --- | --- | --- | --- | --- | --- |
| Ham et al 2018^26^ | Diabetic patients | Korea | conjunctival swab | 16S rRNA sequencing | *Proteobacteria, Firmicutes, Actinobacteria, Cyanobacteria* and *Bacteroidetes*  *Acinetobacter, Burkholderia, Rheinheimera*, and *Micrococcus* in the diabetic group, and *Staphylococcus, Bradyrhizobiaceae, Streptophyta*, and *Novosphingobium* in the healthy subjects. |
| Li Siqi et al 2019^27^ | Type 2 Diabetes Mellitus | Guang Zhou, China | conjunctival swab | 16s rRNA | *Pseudomonas, Acinetobacter, Bacillus and Corynebacterium*. |
| Zhu X. et al 2021^28^ | type 2 diabetes mellitus | Shanghai,  China | Conjunctival swabs | 16s rRNA sequencing | Phlya level: *Proteobacteria, Firmicutes, Actinobacteria*, and *Bacteroidetes*  Genus level: *Staphylococcus, Corynebacterium, enhydrobater, Acinetobacter, Chryseobacterium, Deinococcus, Sphingomonas, streptococcus,* *Pseudomonas* and *Finegoldia* |

**Table S7-VI Microbiome studies in other ocular surface disease: Tumors and proliferation**

| **Author, year** | **Disease** | **Location** | **Sample** | **Sequencing method** | **Species/genus/phylum of commensal** |
| --- | --- | --- | --- | --- | --- |
| Asao K. et al, 2019^29^ | Conjunctival MALT lymphoma | Japan | Conjunctiva, meibomian gland, periocular skin and hand samples | 16S rRNA | *Delftia, Xylophilus, Simplicispira, Rothia* and *Xanthomonas*  Patient group: higher abundance of *Delftia, Clostridium* and *Brevundimonas* and a lower abundance of *Schlegelella* and *Lactobacillus* |

**Figure S1**

**
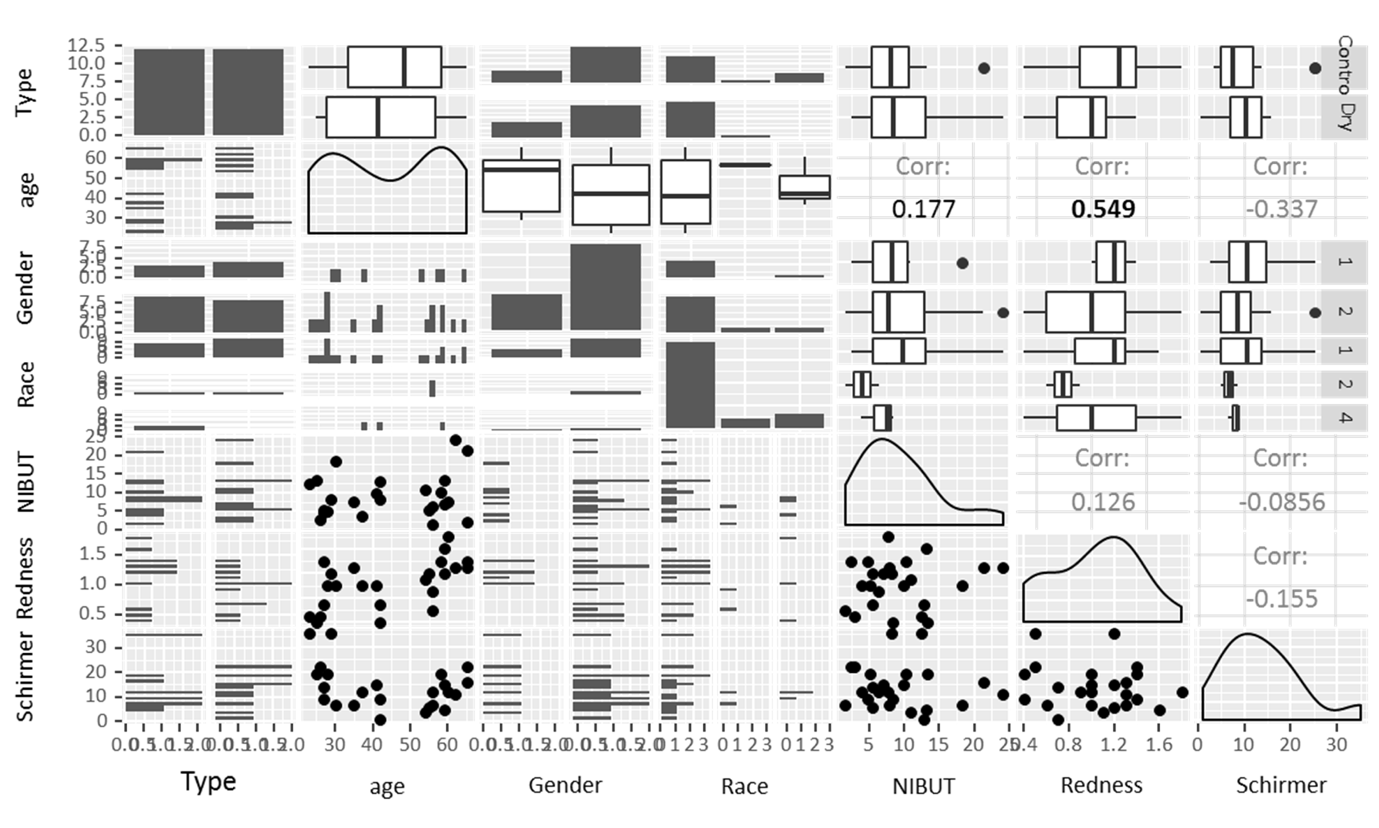
**

**Figure S1:** The association and spread of various clinical and demographic parameters of the participants with each other. For the relationship between continuous and a categorical variable: the display is either box plots or bar charts. For relationship between two continuous variables, either the frequency distribution or the scatter diagrams are shown. Type: dry eye or control participants. Corr: linear correlation coefficient.

**Figure S2**


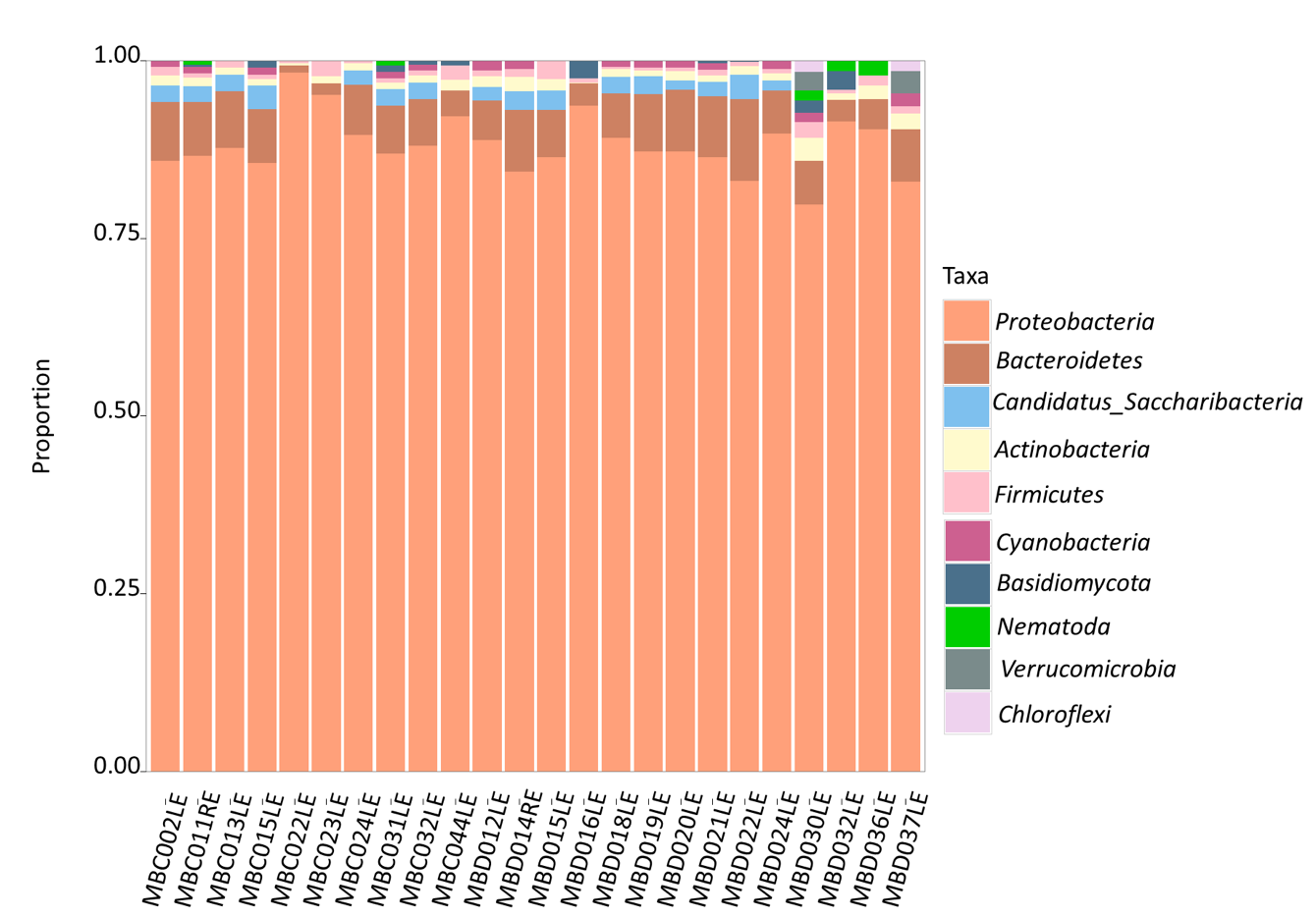


**Figure S2:** The proportion of each major microbial phylum of the total phyla analyzed is indicated as a ratio for each participant (represented by each vertical bar).

**Figure S3**

**
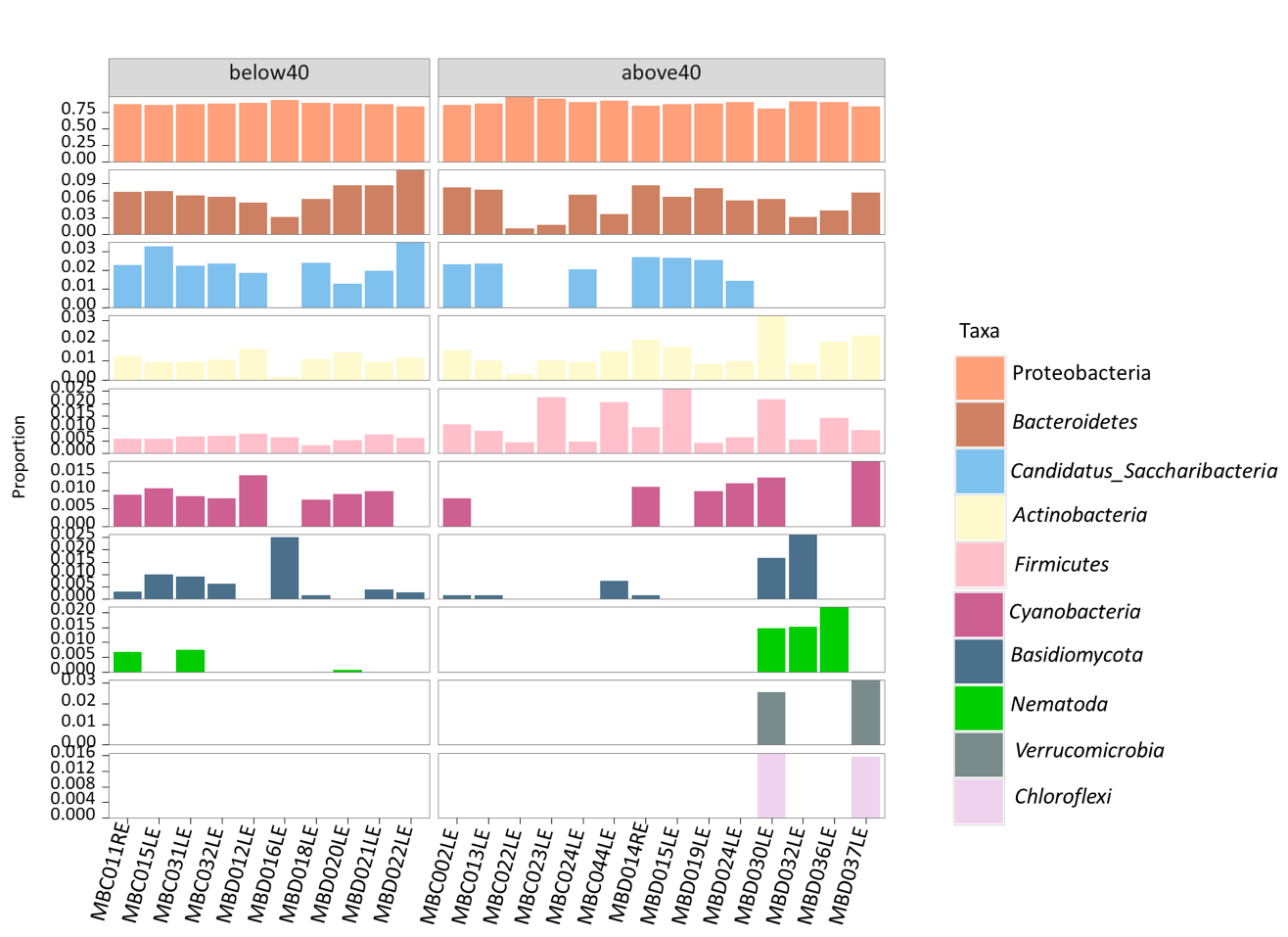
**

**Figure S3:** The relationship of each microbial phylum as a proportion of total phylum (as in Figure S2) with the participants arranged in two categories according to age: above or below 40 years.

**Figure S4**

**
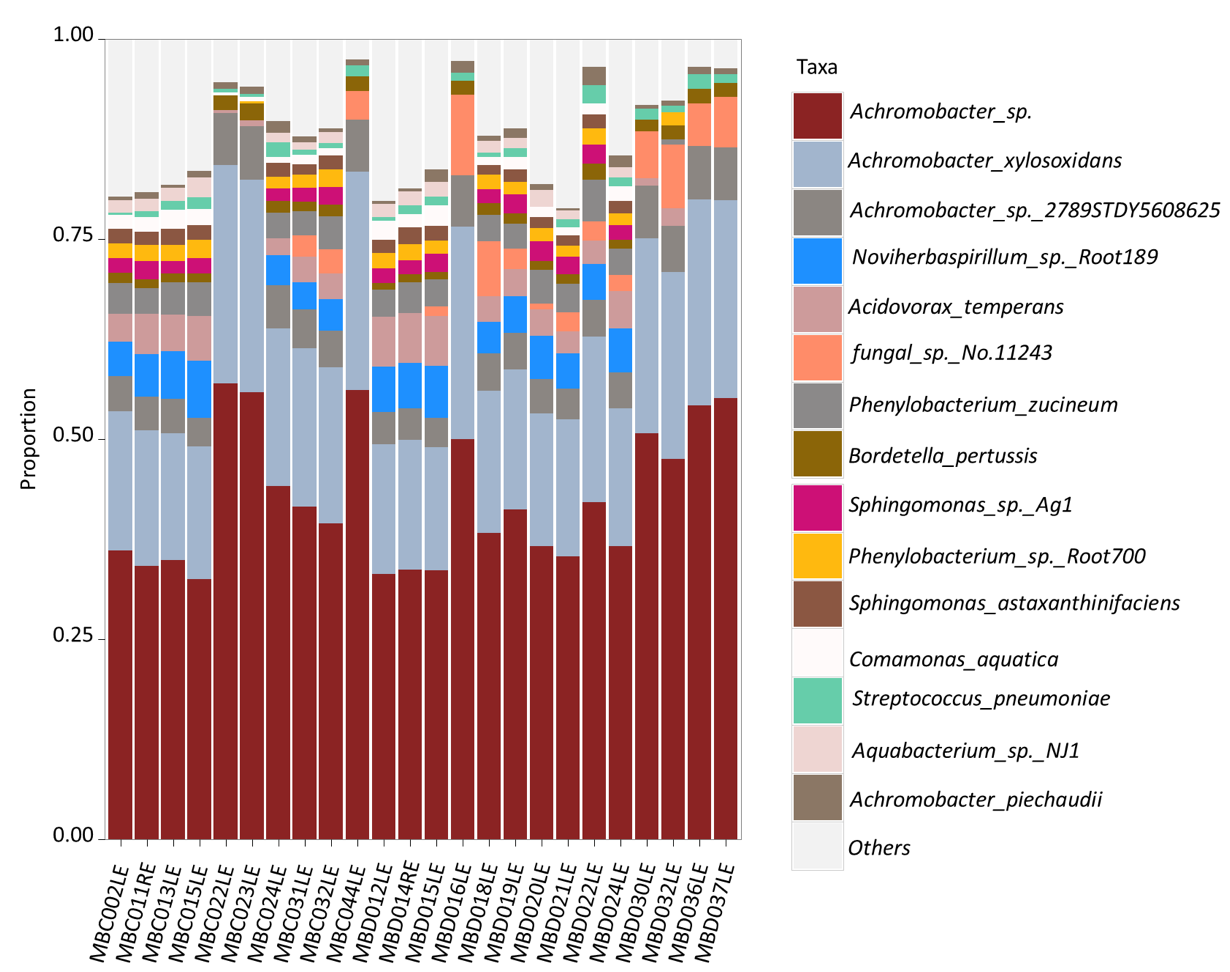
**

**Figure S4:** The proportion of each major microbial specie as a proportion of the total species determined. Each vertical bar represents one participant.

**
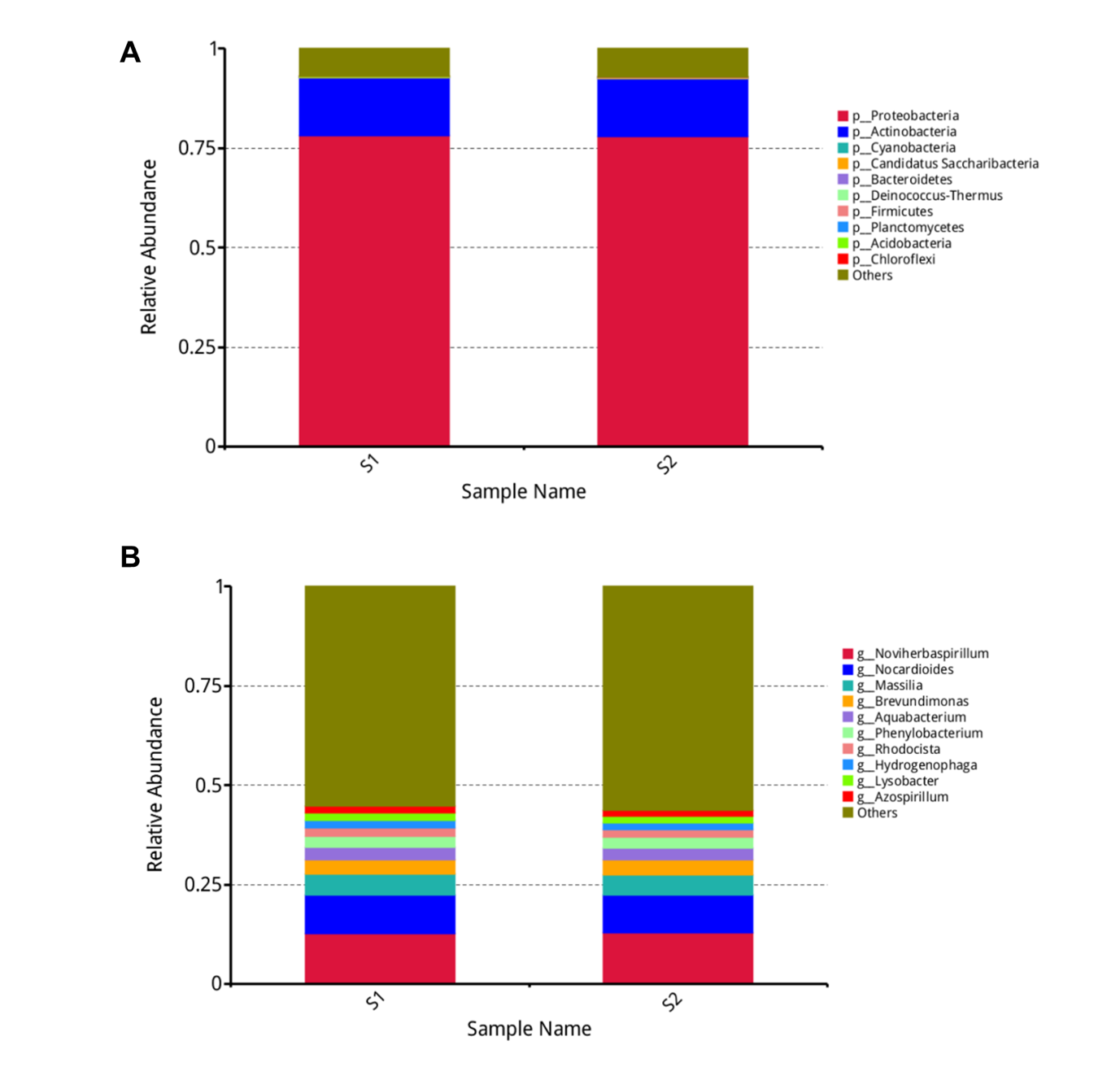
**

**Figure S5** Relative abundance of microbiome from empty swabs control in phylum (A) and genus level (B). An empty swab was an unused swab that was opened under the same room and conditions as the participants and then homogenized and processed as if it has been used on a participant. S1 and S2 are combined samples from three empty swabs. Phylum and genus in “others” are available on request.

**References**

1. Andersson J, Vogt JK, Dalgaard MD, Pedersen O, Holmgaard K, Heegaard S. Ocular surface microbiota in patients with aqueous tear-deficient dry eye. *Ocul Surf*. Jan 2021;19:210-217. doi:10.1016/j.jtos.2020.09.003

2. Willis KA, Postnikoff CK, Freeman A, et al. The closed eye harbours a unique microbiome in dry eye disease. *Sci Rep*. 07 2020;10(1):12035. doi:10.1038/s41598-020-68952-w

3. Li Z, Gong Y, Chen S, et al. Comparative portrayal of ocular surface microbe with and without dry eye. *J Microbiol*. Nov 2019;57(11):1025-1032. doi:10.1007/s12275-019-9127-2

4. Graham JE, Moore JE, Jiru X, et al. Ocular pathogen or commensal: a PCR-based study of surface bacterial flora in normal and dry eyes. *Invest Ophthalmol Vis Sci*. Dec 2007;48(12):5616-23. doi:10.1167/iovs.07-0588

5. de Paiva CS, Jones DB, Stern ME, et al. Altered Mucosal Microbiome Diversity and Disease Severity in Sjögren Syndrome. *Sci Rep*. Apr 2016;6:23561. doi:10.1038/srep23561

6. Zilliox MJ, Gange WS, Kuffel G, et al. Assessing the ocular surface microbiome in severe ocular surface diseases. *Ocul Surf*. 10 2020;18(4):706-712. doi:10.1016/j.jtos.2020.07.007

7. Kittipibul T, Puangsricharern V, Chatsuwan T. Comparison of the ocular microbiome between chronic Stevens-Johnson syndrome patients and healthy subjects. *Sci Rep*. 03 2020;10(1):4353. doi:10.1038/s41598-020-60794-w

8. Sagaser S, Butterfield R, Kosiorek H, et al. Effects of Intense Pulsed Light on Tear Film TGF-β and Microbiome in Ocular Rosacea with Dry Eye. *Clin Ophthalmol*. 2021;15:323-330. doi:10.2147/OPTH.S280707

9. Yun Q, Yong W, Tianhui L, et al. Comparison of the Ocular Microbiomes of Dry Eye Patients With and Without Autoimmune Disease. Frontiers in Cellular and Infection Microbiology; 2021. p. 8.

10. Jiang X, Deng A, Yang J, et al. Pathogens in the Meibomian gland and conjunctival sac: microbiome of normal subjects and patients with Meibomian gland dysfunction. *Infect Drug Resist*. 2018;11:1729-1740. doi:10.2147/IDR.S162135

11. Dong X, Wang Y, Wang W, Lin P, Huang Y. Composition and Diversity of Bacterial Community on the Ocular Surface of Patients With Meibomian Gland Dysfunction. *Invest Ophthalmol Vis Sci*. 11 2019;60(14):4774-4783. doi:10.1167/iovs.19-27719

12. Zhao F, Zhang D, Ge C, et al. Metagenomic Profiling of Ocular Surface Microbiome Changes in Meibomian Gland Dysfunction. *Invest Ophthalmol Vis Sci*. Jul 1 2020;61(8):22. doi:10.1167/iovs.61.8.22

13. Lee SH, Oh DH, Jung JY, Kim JC, Jeon CO. Comparative ocular microbial communities in humans with and without blepharitis. *Invest Ophthalmol Vis Sci*. Aug 15 2012;53(9):5585-93. doi:10.1167/iovs.12-9922

14. Yan Y, Yao Q, Lu Y, et al. Association Between Demodex Infestation and Ocular Surface Microbiota in Patients With Demodex Blepharitis. *Front Med (Lausanne)*. 2020;7:592759. doi:10.3389/fmed.2020.592759

15. Chao C, Akileswaran L, Cooke Bailey JN, et al. Potential Role of Ocular Microbiome, Host Genotype, Tear Cytokines, and Environmental Factors in Corneal Infiltrative Events in Contact Lens Wearers. *Invest Ophthalmol Vis Sci*. 12 2018;59(15):5752-5761. doi:10.1167/iovs.18-24845

16. Shin H, Price K, Albert L, Dodick J, Park L, Dominguez-Bello MG. Changes in the Eye Microbiota Associated with Contact Lens Wearing. *mBio*. Mar 22 2016;7(2):e00198. doi:10.1128/mBio.00198-16

17. Yau JW, Hou J, Tsui SKW, et al. Characterization of ocular and nasopharyngeal microbiome in allergic rhinoconjunctivitis. *Pediatr Allergy Immunol*. 09 2019;30(6):624-631. doi:10.1111/pai.13088

18. Liang Q, Li J, Zhang S, et al. Characterization of conjunctival microbiome dysbiosis associated with allergic conjunctivitis. *Allergy*. 02 2021;76(2):596-600. doi:10.1111/all.14635

19. Vishwakarma P, Mitra S, Beuria T, Barik MR, Sahu SK. Comparative profile of ocular surface microbiome in vernal keratoconjunctivitis patients and healthy subjects. *Graefes Arch Clin Exp Ophthalmol*. Mar 2021;doi:10.1007/s00417-021-05109-z

20. Butcher RMR, Sokana O, Jack K, et al. Active Trachoma Cases in the Solomon Islands Have Varied Polymicrobial Community Structures but Do Not Associate with Individual Non-Chlamydial Pathogens of the Eye. *Front Med (Lausanne)*. 2017;4:251. doi:10.3389/fmed.2017.00251

21. Pickering H, Palmer CD, Houghton J, et al. Conjunctival Microbiome-Host Responses Are Associated With Impaired Epithelial Cell Health in Both Early and Late Stages of Trachoma. *Front Cell Infect Microbiol*. 2019;9:297. doi:10.3389/fcimb.2019.00297

22. Shivaji S, Jayasudha R, Chakravarthy SK, et al. Alterations in the conjunctival surface bacterial microbiome in bacterial keratitis patients. *Exp Eye Res*. 02 2021;203:108418. doi:10.1016/j.exer.2020.108418

23. Cavuoto KM, Galor A, Banerjee S. Ocular Surface Microbiome Alterations Are Found in Both Eyes of Individuals With Unilateral Infectious Keratitis. *Translational Vision Science & Technology*. 2021;10(2):19-19. doi:10.1167/tvst.10.2.19

24. Kang Y, Zhang H, Hu M, et al. Alterations in the Ocular Surface Microbiome in Traumatic Corneal Ulcer Patients. *Invest Ophthalmol Vis Sci*. 06 2020;61(6):35. doi:10.1167/iovs.61.6.35

25. Ge C, Wei C, Yang BX, Cheng J, Huang YS. Conjunctival microbiome changes associated with fungal keratitis: metagenomic analysis. *Int J Ophthalmol*. 2019;12(2):194-200. doi:10.18240/ijo.2019.02.02

26. Ham B, Hwang HB, Jung SH, Chang S, Kang KD, Kwon MJ. Distribution and Diversity of Ocular Microbial Communities in Diabetic Patients Compared with Healthy Subjects. *Curr Eye Res*. 03 2018;43(3):314-324. doi:10.1080/02713683.2017.1406528

27. Li S, Yi G, Peng H, et al. How Ocular Surface Microbiota Debuts in Type 2 Diabetes Mellitus. *Front Cell Infect Microbiol*. 2019;9:202. doi:10.3389/fcimb.2019.00202

28. Zhu X, Wei L, Rong X, et al. Conjunctival Microbiota in Patients With Type 2 Diabetes Mellitus and Influences of Perioperative Use of Topical Levofloxacin in Ocular Surgery. *Front Med (Lausanne)*. 2021;8:605639. doi:10.3389/fmed.2021.605639

29. Asao K, Hashida N, Ando S, et al. Conjunctival dysbiosis in mucosa-associated lymphoid tissue lymphoma. *Sci Rep*. 06 2019;9(1):8424. doi:10.1038/s41598-019-44861-5
